# Pleistocene climatic oscillations in Neotropical open areas: Refuge isolation in the rodent *Oxymycterus nasutus* endemic to grasslands

**DOI:** 10.1371/journal.pone.0187329

**Published:** 2017-11-27

**Authors:** Willian T. Peçanha, Sergio L. Althoff, Daniel Galiano, Fernando M. Quintela, Renan Maestri, Gislene L. Gonçalves, Thales R. O. Freitas

**Affiliations:** 1 Programa de Pós-Graduação em Genética e Biologia Molecular, Universidade Federal do Rio Grande do Sul, Rio Grande do Sul, Brazil; 2 Departamento de Ciências Naturais, Laboratório de Biologia Animal, Universidade Regional de Blumenau, Blumenau, SC, Brazil; 3 Pós-graduação em Ciências Ambientais, Area de Ciências Exatas e Ambientais, Unochapecó, Santa Catarina, Brazil; 4 Programa de Pós-Graduação em Biologia de Ambientes Aquáticos Continentais, Instituto de Ciências Biológicas, Universidade Federal do Rio Grande, Rio Grande do Sul, Brazil; 5 Programa de Pós-Graduação em Biologia Animal, Departamento de Zoologia, Universidade Federal do Rio Grande do Sul, Rio Grande do Sul, Brazil; 6 Departamento de Recursos Ambientales, Facultad de Ciencias Agronómicas, Universidad de Tarapacá, Arica, Chile; Universita degli Studi di Roma La Sapienza, ITALY

## Abstract

Pleistocene climatic oscillations favoured the expansion of grassland ecosystems and open vegetation landscapes throughout the Neotropics, and influenced the evolutionary history of species adapted to such environments. In this study, we sampled populations of the rodent *Oxymycterus nasutus* endemic to open areas in the Pampas and Atlantic Forest biomes to assess the tempo and mode of population divergence using an integrative approach, including coalescence theory, ecological niche models, and morphometry. Our results indicated that these *O*. *nasutus* populations exhibited high levels of genetic structure. Six major mtDNA clades were found, structuring these biomes into distinct groups. Estimates of their divergence times was indicated to be 0.571 myr. The high degree of genetic structure is reflected in the analyses of geometric morphometric; skull differences between lineages in the two ecoregions were detected. During the last glacial maximum, there was a strong increase in suitable abiotic conditions for *O*. *nasutus*. Distinct molecular markers revealed a population expansion over time, with a possible demographic retraction during the post-glacial period. Considering that all clades coalesce with the last interglacial maximum, our results indicated that reduction in suitable conditions during this period may have resulted in a possible vicariance associated with refuge isolation.

## Introduction

The glaciations occurred in the Pleistocene (2.58–0.01 million years ago [myr]), a period of climatic history that is known with near accuracy [1], which led to the migrations and reductions in the population sizes of several species, followed by recolonization and population expansion as glaciers retreated [2–4]. Therefore, the divergence promoted by glaciers have probably affected populations distributed in allopatry, which could be detected in genealogies, as observed in typical glacial refuges [5].

In the recent years, some regions in the Neotropics have been systematically studied [6], as the high biological diversity provides an opportunity to test refuge hypotheses associated with climatic oscillations in the Quaternary Epoch, especially in complex ecoregions such as the Atlantic Forest [7,8]. A remarkable landscape in South America are the open areas, such as the Pampas biome, the largest temperate grasslands in the world [9], which are excellent environments to evaluate the influence of historical climatic fluctuations on the origin and evolutionary dynamics of species. The cool and dry weather during the glaciations allowed grassland ecosystems and open vegetation landscapes to expand over much of South America [10], which created new ‘suitable’ areas. Thus, it is expected that a species endemic to open areas respond to Pleistocene climatic oscillations in a different way than a species adapted to forest environments, [11–15], particularly, those that occupy heterogeneous grasslands ecoregions such as the Akodontine rodent *Oxymycterus nasutus* (Waterhouse, 1837).

**Fig 1:**
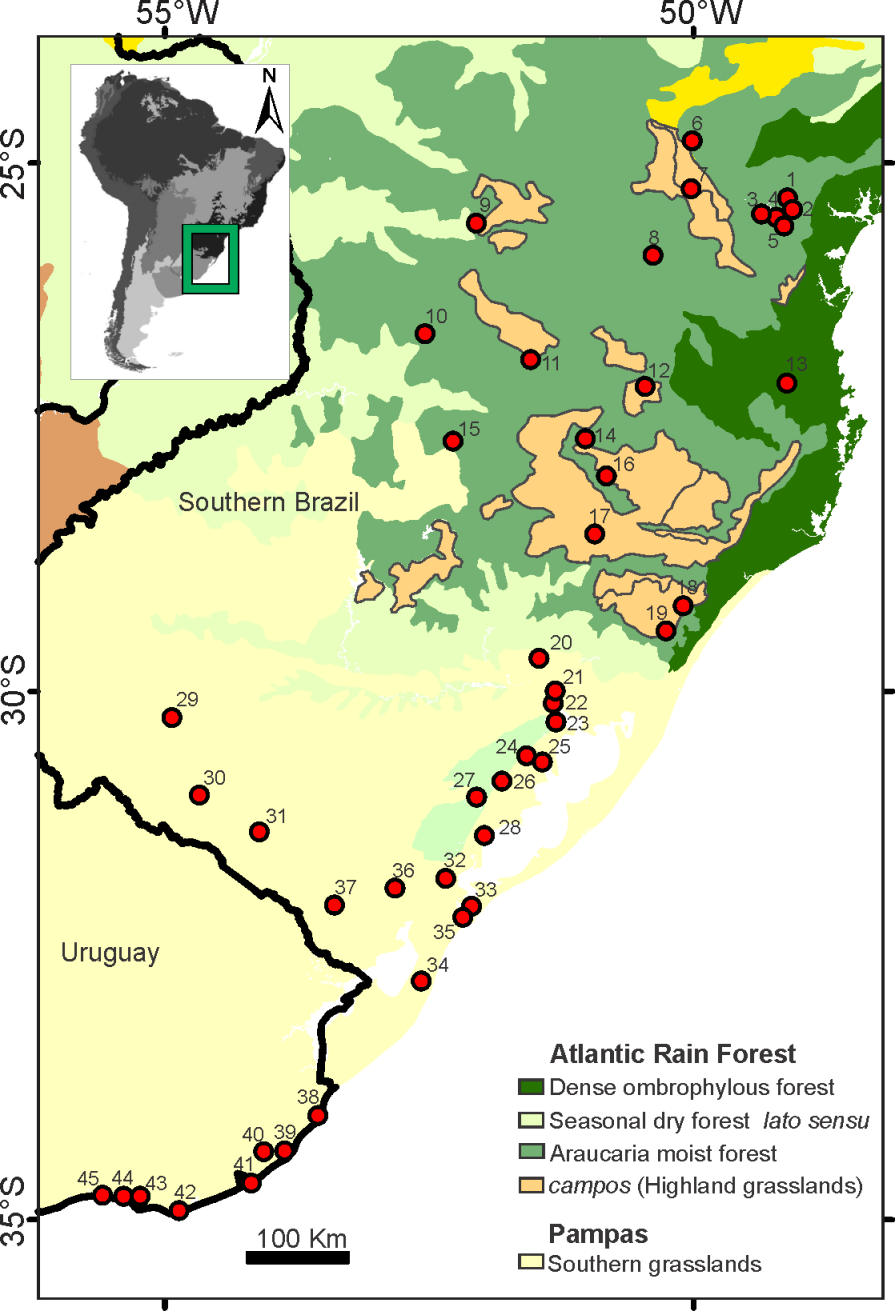
Geographic distribution of *O*. *nasutus* and sampling sites in Southern Brazil and Uruguay. The map includes the ecoregion where the species is located. Details of site numbers are found in the Table 1. The map were obtained from Ecoregions2017Resolve (available at: http://ecoregions2017.appspot.com/). Any rights in individual contents of the database are licensed under the Database Contents License: https://creativecommons.org/licenses/by/4.0/), and edited with ArcMap 10.3 software.

*O*. *nasutus* is an abundant species adapted to wet areas, thriving at sea level in the southernmost Uruguay and Rio Grande do Sul State, Brazil, to higher elevations (up to 900 m) on the Santa Catarina and Parana States, Brazil, where it is restricted by the coastal mountains [16–17] (Fig 1). It inhabits coastal sandbanks, wet grasslands, steppes, and other phytophysiognomies of the Pampas, a biome that dominate Uruguay and the plains of southernmost Brazil. Moreover, it is highly dispersed throughout the southern Brazilian plateau in high-elevation field domains, and is probably the most abundant small mammal in the high-elevation grasslands in the states of Rio Grande do Sul and Santa Catarina (Atlantic Forest domain) [17–19].

The relationship between the grasslands and the forests during the late Quaternary is well documented in the studies on pollen records in the South and Southeast Brazil [20, 10]. Overall, general patterns of grasslands distributions up to the last glacial period in the Pleistocene (42,000–10,000 years BP) is linked with the rise of vegetation formations in the late Holocene [21]. In Southern Brazil, the expansion of grasslands was promoted by cold weather conditions and dry climate owing to the glaciation periods, with a mean decrease of temperature between 5–7°C in high-altitude (Meridional Plateau) areas [10, 21]. In addition, during the Last Glacial Maximum ([LGM] ~21,000 years BP), grasslands were also present abundantly in the coastal lowlands and on the exposed continental shelf, where forests were almost absent [22–23,10].

Since *O*. *nasutus* is intrinsically related to the grasslands and is abundantly found in these habitats [17–19], cyclic events of the dynamic expansion and retraction of open areas and forests that occurred during glacial and interglacial periods have probably influenced the evolutionary history of this species. Accordingly, to understand the tempo and mode of population divergence and to contribute insights into the aspects of the biogeographic history of the South America, we analysed the phylogeography, population genetics, and skull morphometry across the entire distributional range. We characterized the geographical patterns of genetic and morphological variation and used these data to investigate whether glacial and interglacial periods influenced the extant distribution of *O*. *nasutus* in distinct biomes, hypothesizing the existence of significant divergence between Pampas and Atlantic Forest lineages. Additionally, we estimated the current and historical potential distribution area of *O*. *nasutus* based on paleoclimatic models to allow a detailed evaluation of the demographic history and phylogeographical patterns.

## Materials and methods

### Ethics statement

Skull and tissue samples of *O*. *nasutus* surveyed in the study were obtained from the specimens deposited in the scientific collections (Museo Nacional de Historia Natural [MNHN], Museu de História Natural Capão da Imbuía [MHNCI], Museu de História Natural of the Universidade Luterana do Brasil [MCNU], Fundação Zoobotânica-Museu de Ciências Naturais [FZB-MCN], Universidade Federal do Paraná [UFPR] and Universidade Regional de Blumenau [FURB]), who kindly lent the specimens for use in this study. Specimens deposited in the Universidade Federal do Rio Grande do Sul [UFRGS] were collected (previously) by our research group with the license issued by the Brazil Government authorities.

### Sample collection

A total of 135 specimens of *O*. *nasutus* from 45 localities covering the entire known distribution of this species (Fig 1; Table 1) were surveyed. Field collection (permits granted by the Ministério do Meio Ambiente, Brazil—SISBIO 30204–1, 52650–1 and 29358–1) followed the guidelines of the American Society of Mammalogists for the use of wild mammals in research (https://www.mammalsociety.org/committees/animal-care-and-use). Specimens were euthanized via overdoses of isofluorane and cervical dislocation, procedures authorized by The Animal Care and Use Committee of the Institute of Biological Sciences at UFRGS, Brazil, for this study Specimens trapped were deposited both in the mammal collections of UFRGS and MCNU.

**Table 1:** Sampling localities of *Oxymycterus nasutus* in Southern Brazil and Uruguay.

					Haplotype			
#	Sample site^a^	Lat. (S), Long. (W)	N	Voucher^b^	*Cytb*	*Fgb*-I7	Haplogroup	Skull
1	BR: PR, Quatro Barras	-25.3658, -49.0769	4	MHNCI 4605,4607,4608,4595	H1	H3, H15, H24	Eastern	+
2	BR: PR, Piraquara	-25.4419, -49.0627	8	UFPR-P42,53,54,56,59,60,62,65	H1-H5	H3, H17	Eastern	-
3	BR: PR, Curitiba	-25.4789, -49.3307	1	UFPR-P86	H1	-	Eastern	-
4	BR: PR, Curitiba, Parque Regional do Iguaçu	-25.5229, -49.2229	1	MHNCI 3433	H1	H1	Eastern	+
5	BR: SC, São José dos Pinhais	-25.5799, -49.1753	11	UFPR-P969, 995*,1008*,1018*,1019*,1039*,1049*,1051*,1053*,1055,1061	H1	H3, H18, H19	Eastern	+
6	BR: SC, Castro	-24.7908, -50.0120	4	MHNCI 816–818, 0821	-	-	Central	+
7	BR: SC, Ponta Grossa	-25.2440, -50.0227	7	UFPR-P76, MHNCI 642, 657, 709, 723, 838, 839	H6	-	Central	+
8	BR: SC, São Mateus do Sul	-25.8738, -50.3827	1	MHNCI 3192	H7	-	Northwest	+
9	BR: SC, Candói,	-25.5708, -52.0527	1	CZFURB 18228	H8	H1; H2	Northwest	+
10	BR: SC, São Domingos	-26.6163, -52.5388	2	CZFURB 18119, 18153	H11	H8, H11, H12	Central	+
11	BR: SC, Agua Doce	-26.9977, -51.5558	2	CZFURB 9365, 9856	H12, H13	H1, H3-5	Central	+
12	BR: SC, Ponta Alta do Norte	-27.1153, -50.4577	2	MHNCI 4951, 4596	H9, H10	H1; H10	Northwest	+
13	BR: SC, Indaial	-27.0830, -49.1166	1	CZFURB 9825	H10	H15; H15	Central	+
14	BR: SC, Abdon Batista	-27.6108, -51.0227	1	CZFURB 20520	H2	H13; H14	Central	+
15	BR: RS, Erechim	-27.6338, -52.2738	2	CMLCE-UFRGS HFE 2, 4	H2	H1; H6, H7	Central	-
16	BR: RS, Campo Belo do Sul	-27.9625, -50.8231	4	CZFURB 15106*, 15109, 15140, 15154*	H2	H8; H9	Central	+
17	BR: RS, Vacaria	-28.511944, -50.933889	1	MCNU 2498	H2	-	Central	-
18	BR: RS, Cambará do Sul	-29.191667, -50.0975	2	CMLCE-UFRGS AS5, 17	H14	H1, H14, H20	Eastern	-
19	BR: RS, São Francisco de Paula	-29.428322, -50.259444	10	MCNU 3043, 3210, 3658, 3656*, 3657*; CMLCE-UFRGS PM 100, 104, 74, 79, 86	H14, H15	H1, H8, H16-17, H21-24	Eastern	+
20	BR: RS, Montenegro	-29.682555, -51.466450	1	FZB-MCN 547	-	-	Steppes Plain	+
21	BR: RS, Eldorado do Sul	-29.997139, -51.307861	1	FZB-MCN 675	-	-	Steppes Plain	+
22	BR: RS, Guaíba	-30.113889, -51.325	11	MCNU 3211, 3652, 3228, 3119, 3009, 3141, 3146*, 3142, 3149, 3212, 3653	H16	H1, H3, H24, H26, H33-34	Steppes Plain	+
23	BR: RS, Barra do Ribeiro	-30.290833, -51.300833	6	MCNU 3144, 3654*, 3147, 3230, 3039, 3135	H16, H17, H18	H1, H33-35	Steppes Plain	+
24	BR: RS, Sentinela do Sul	-30.610833, -51.578889	1	MCNU 314	H19	H1; H29	Steppes Plain	+
25	BR: RS, Tapes	-30.669849, -51.429707	1	MCNU 3132	H19	-	Steppes Plain	+
26	BR: RS, Camaquã	-30.850833, -51.811944	10	CMLCE-UFRGS FQ 47; MCNU 3011, 3012, 3110, 3116, 3122, 3124, 3133, 3214, 3227; CZFURB 6249*,6250	H20-H24	-	Steppes Plain	+
27	BR: RS, Cristal	-31.002778, -52.050	3	MCNU 4331; CMLCE-UFRGS FQ 63, 72	H22	H1; H30		+
28	BR: RS, São Lourenço do Sul	-31.365, -51.977778	5	MCNU 3225, 3109, 3123, 3115, 3010	H19, H26	H1, H26; H31	Steppes Plain	+
29	BR: RS, Rosário do Sul	-30.247938, -54.924036	1	FZB-MCN 648	-	-	Steppes Plain	+
30	BR: RS, Dom Pedrito	-30.975882, -54.666567	2	FZB-MCN 710, 1011	-	-	Steppes Plain	+
31	BR: RS, Bagé	-31.330833, -54.106944	2	CMLCE-UFRGS ALL 12, 13	H25	H1, H25-27	Steppes Plain	-
32	BR: RS, Pelotas	-31.771944, -52.342778	4	CMLCE-UFRGS PL 300; MCNU 3223, 3041,3042	H19, H27	H1, H28-30	Steppes Plain	+
33	BR: RS, Rio Grande	-32.035, -52.098889	2	CMLCE-UFRGS MEV 01; MCNU 3014	H31	H31	Taim Wetland	-
34	BR: RS, Rio Grande, ESEC Taim	-32.7425, -52.574444	3	MCNU 3661*, 3131, 3660	H32, H33	H31	Taim Wetland	+
35	BR: RS, Rio Grande, ÁPA Lagoa Verde	-32.139934, -52.181064	1	MCNU 3660	H33	-	Taim Wetland	+
36	BR: RS, Pedro Osório	-31.863889, -52.822778	4	CMLCE-UFRGS POS 18, 20, 25, 27	H28, H29	H26; H36	Southern	-
37	BR: RS, Herval	-32.023889, -53.395833	1	CMLCE-UFRGS HL 01	H30	H31; H37	Southern	-
38	UY: Rocha, Parque Santa Teresa	-34.008180, -53.552735	1	MNHN SN EMG1809	-	H3; H3	Southern	-
39	UY: Rocha, Laguna de Castillos	-34.35, -53.866667	2	MNHN SN SCV 108, 110	H34	H38, H29; H30 H39	Southern	-
40	UY: Rocha, Route 9 km 304.800	-34.357743, -54.064845	1	MNHN SN GD 577**	H35	-	Southern	-
41	UY: Rocha, La Paloma, La Palma	-34.655896, -54.181969	2	MNHN SN CA 614, 617*	H34	H24; H24	Southern	+
42	UY: Maldonado, San Carlos	-34.915632, -54.865456	2	MNHN SN GD 723; MVZ 182701 (CA458)**	H37, H38	H3; H31	Southern	-
43	UY: Maldonado, Pan de Azucar	-34.779218, -55.232399	1	MNHN SN CA 680*	-	-	Southern	+
44	UY: Maldonado, Solís Grande	-34.783273, -55.334011	1	MNHN SN CA 695**	H37	-	Southern	+
45	UY: Canelones, La Floresta	-34.770278, -55.588333	1	MNHN 5615 (EMG1567)	H36	H26; H31	Southern	-

Localities (#) are mapped in Fig 1. Vouchers, Cytb/Fgb-I7 haplotypes and presence (+)/absence (-) of skull samples per site are presented.

^a^: Abreviations: BR: Brazil, UY: Uruguay; PR, Paraná; SC, Santa Catarina; RS, Rio Grande do Sul states.

^b^: Acronyms of collections: UFPR-P, Scientific Collection of the Cytogenetic and Conservation Laboratory at the Universidade Federal do Paraná; MHNCI, Museu de História Natural Capão da Imbuía; CZFURB, Zoological Collection of the Universidade Regional de Blumenau; CMLCE-UFRGS, Mastozoological Collection of the Cytogenetic Laboratory and Evolution at the Universidade Federal do Rio Grande do Sul; MCNU, Museu de História Natural of the Universidade Luterana do Brasil; MNHN, Museo Nacional de Historia Natural (Uruguay); FZB-MCN, Fundação Zoobotânica-Museu de Ciências Naturais; MVZ, Museum of Vertebrate Zoology at the University of California/Berkeley.

*Vouchers presenting only skulls.

**Data obtained from NIH genetic sequence database (Genbank; https://www.ncbi.nlm.nih.gov/genbank/)

### Phylogenetic relationships

DNA was isolated from 107 specimens using the PureLink Genomic DNA extraction kit (Invitrogen, Life Technologies), following the manufacturer's instructions. Polymerase chain reaction (PCR) was performed to amplify the mitochondrial DNA (mtDNA) cytochrome *b* (*Cytb*) gene (801 bp) and the nuclear locus beta-fibrinogen, intron 7 (*Fgb-*I7) (408 bp) according to the method described by Smith and Patton [24] and Matocq et al. [25]. The nuclear marker was chosen as it represents a single-copy locus, and is informative and evolving at a different rate compared to the mtDNA [26]. PCR products were stained with GelRed (Biotium) and checked on 1% agarose gel, purified with enzymatic method (Exonuclease and Alkaline Phosphatase; Amersham Biosciences, Piscataway, NJ) and sequenced with Sanger method using both primers (forward and reverse). All sequences obtained were deposited in GenBank under the accession numbers: *Cyt b*, MF766110 to MF766188 and *Fgb*-I7, MF766189 to MF766256.

Alignment and editing of the mtDNA *Cytb* sequences was performed in the Clustal W algorithm using the MEGA 7 software. For the nuclear *Fgb-I7*, sequences were aligned using MAFFT v.7.245 [26] with the auto setting. Variable sites of this nuclear marker were assessed in the original chromatograms to ensure correct identification of the heterozygotes. Heterozygous sites were identified when two different nucleotides were indicated at the same position in the chromatograms, with the weakest peak reaching at least 25% of the strongest signal. The IUPAC symbols were applied for coding the double peaks. The gametic phase of each haplotype was identified computationally using the program PHASE v2.1 [27, 28] as implemented in DnaSP v5.10 [29] to reconstruct putative alleles of the nuclear marker for use in downstream haplotype-based analyses. The program was ran with 500 burn-in steps and 500 iterations, including the allowed intragenic recombination for the given data set. We also used a 0.6 output probability threshold for haplotypes and genotypes, as this was shown to reduce the number of unidentified genotypes with or without slight increase in the number of false positives [30].

The most appropriate substitution model for mtDNA phylogeny from the haplotypic data was the HKY+G that was determined based on the Akaike Information Criterion in the jModelTest v2.1.10 software [31]. The phylogeny was reconstructed using Bayesian Inference (BI) in the software BEAST 1.8.4 [32]. Two independent Markov Chain Monte Carlo (MCMC) runs, each with four streams per 25 million steps of the MCMC, sampled every 1000 generations, and discarding 2.5 million burn-in (about 10% trees discarded), starting the initial trees with randomness, without restriction were performed. Speciation tree prior was modelled on Birth-Death Process using the BEAST software. Parameter convergence was checked in Tracer v1.5 [33] as to whether effective sample sizes (ESS) reached 200. The remaining trees were used to calculate the posterior probabilities for each node.

The burn-in was determined in Tracer v1.6 based on the trajectory parameters, and 10% of the initial trees were removed and summarized in TreeAnnotator. The consensus tree generated was visualized and edited in Figtree v1.4.3 (http://tree.bio.ed.ac.uk/software/figtree/). There were no fossils for *Oxymycterus* that could be used for a specific estimate of the substitution rate. Hence, we used an evolutionary rate already published [34] of 2.37% per million years (Myr^-1^) for the genus. Accordingly, the divergence times were estimated in BEAST assuming an uncorrelated relaxed clock model and a normal distribution for the substitution rate, with a mean of 2.37% Myr-1, and a standard deviation of 0.25% Myr-1, to allow some uncertainty in the evolutionary rate. One representative sequence for each haplotype was used. Sequences of *Oxymycterus delator* (U03525), *Oxymycterus dasytrichus* (AF454768), *Oxymycterus amazonicus* (AF454765), and sister lineages of *O*. *nasutus* [35], were included in the analysis. The time of the most recent common ancestor (TMRCA) for relevant nodes and major mitochondrial clades was reported as the mean value of node height with 95% highest posterior density interval (HPD). The nodes were supported with the posterior probability (PP) of ≥ 90% [36]. We also reconstructed the phylogenetic tree among nuclear DNA (nDNA) haplotypes (Fgb-I7) data set with phased alleles and single-copy sequences using BI in BEAST. However, due to the low variability and lack of support, we performed downstream analysis based on the haplogroups inferred by mtDNA. The topology obtained from *Fgb*-I7 evolutionary tree is shown in S1 Fig.

The evolutionary relationships between the haplotypes for each data set (i.e. mtDNA and nDNA) were estimated using the median-joining method implemented in the Network v5.0 [37] (http://www.fluxus-engineering.com). For assessing the evolutionary relationships of the *Fgb*-I7 gene, haplotypes were identified by coalescent-based Bayesian method implemented in PHASE v2.1. Missing/gaps sites were verified and invariant sites were removed from the dataset. Indels were treated as single mutational events.

### Intraspecific variation and historical demography

Statistical parameters of diversity, such as the number of variable sites (S), number of haplotypes (h), haplotype diversity (Hd), nucleotide diversity (π), and average number of nucleotide differences (k) were measured using DNASP v5.10 [29]). We calculated the genetic distance between the recognized haplogroups of *Cytb* (average distances) using p-distance model with 1000 bootstrap replications using MEGA v7 [38]. The defined clades were assumed based on the phylogeny obtained through Bayesian analysis.

Historical demographic changes such as signatures of demographic expansion, and equilibrium or decline were examined for the species and for each haplogroup using neutrality tests, such as Tajima’s D [39] and Fu’s Fs [40] statistics, employing the software DNASP v5.10. Pairwise mismatch distribution was performed in DNASP v.5.10 to infer the historical demography of *O*. *nasutus*, calculated with the expected frequency based on a population growth-decline model. The sum of squared deviations (SSDs) between the observed and expected mismatch distribution and the raggedness index (r) were calculated to test the null hypothesis of spatial expansion using ARLEQUIN v3.5 [41]. In addition, we performed a Bayesian skyline plot (BSP) analysis, which does not assume a priori any growth model and infers the effective population size through time based on coalescent theory [42]. The BSP was used to estimate the dynamics of alterations in the overall population size over time for the *O*. *nasutus* dataset. BEAST v1.8.4 [32] was used for estimating the BSP of mtDNA as described above, with minor alterations in the number of parameters sampled at every 10,000 steps. For nDNA, a posterior distribution model of effective population size through time was generated using a MCMC sampling scheme. Two independent analyses were ran for 40 x 10^7^ generations (sampling at every 10,000 and 10% burn in) under a HKY+G substitution model (obtained through the jModelTest 2 software), assuming a relaxed molecular clock model. However, we used the substitution rates and dates estimated from previous studies to calibrate a relaxed molecular clock and approximate divergence times for the main phylogroups retrieved in our study. We implemented a lognormal prior distribution based on the substitution rate for the cricetid genus *Peromyscus* (0.006 ± 0.003 substitution/site/million years) for the *Fgb*-I7 data set [43]. Skyline reconstruction was performed in Tracer v1.5, and the median and 95% credibility interval were plotted as a function of time.

### Ancestral area reconstruction

Aiming to reconstruct the ancestral range of *O*. *nasutus*, we performed a Statistical dispersal–vicariance analysis (S–DIVA) on the maximum clade credibility tree generated from the BEAST analysis using the software RASP 3.02 [44–46]. The DIVA method [44] develops an approach based on parsimony events and reconstructs ancestral distributions based on a simple biogeographic model and a three-dimensional cost matrix. Penalties are not assigned when speciation is the result of vicariance; however, dispersal and extinction events have a penalty of one per unit area added to or deleted from a distribution. S-DIVA method rectified the problems in DIVA analysis and suggested possible ancestral ranges at each node and also calculated the probabilities for each ancestral range at the nodes. Due to the current area of occurrence and based on historical aspects of the region (expansion and retreat of forest formations), we reconstructed the ancestral areas only within the Pampas (A) and Atlantic Forest/grassland mosaic (B) regions. According to the results obtained by BI and the presence of restricted haplogroups in a single ecoregion, the clades recognized in *O*. *nasutus* were categorized in these areas. We used the output files generated by BEAST (total and condensed trees) for the analysis.

### Morphological analyses

Digital photographs were taken from the dorsal, lateral, and ventral views of the skull of 89 specimens of *O*. *nasutus* (Table 1). Only adult specimens were photographed to minimize the ontogenetic effects (the complete eruption of the third molar was the criterion to separate juveniles from adults). Two-dimensional digital images were taken using a Canon PowerShot G10 camera with 14.7 megapixels resolution (4416 x 3312) in the macro function of the automatic mode and without flash or zoom. The pictures were taken from a standard distance of 127 mm for all the specimens. A total of 17 landmarks were digitized in the dorsal view, 16 landmarks in the lateral view, and 32 landmarks in the ventral view (S2 Fig), using the TpsDig2 software [47]. The same person (WTP) conducted the digitization. The choice of landmarks was based on previous studies with sigmodontines [48,49]. In the dorsal and ventral view, all individuals were marked on both sides of skull. The description of all the landmarks employed is given in S1 Appendix. After digitization, a Generalized Procrustes Analysis (GPA) was conducted on the matrix of landmark coordinates to remove the effects of scale, position, and orientation [50]. The centroid size, derived by the square root of the sum of squared distances of each landmark from the centroid of the configuration [51], was used as a measure of size. We appended the natural log-transformed centroid size into the matrix of shape coordinates to work in the form of space for further analyses.

Our goal was to investigate whether morphology is divergent among the haplogroups found in the genetic analyses (Northwest, Central, Eastern, Steppes Plain, Southern, and Taim Wetland), and also between physiognomies or “environmental groups” (Pampas versus Atlantic Forest). First, we conducted a principal component analysis (PCA) in the form matrix for each view. The number of PCs necessary to achieve 100% variation in each view (16 PCs for dorsal view, 31 PCs for ventral view, and 29 PCs for lateral view) were used as response variables for downstream analyses. We performed multivariate analyses of variance (MANOVA) and discriminant analyses with Jackknife cross validation (DA) to investigate how morphology was structured among the six genetic haplogroups (first predictor) and the two environmental groups (second predictor). MANOVAs and DAs were performed independently for each predictor and for each skull view. All procedures were carried out in the software R [52]), with the packages geomorph [53] and Morpho [54]. Visualization of shape changes was made by comparing the shapes among groups using discriminant functions implemented in MorphoJ [55].

### Geographic distribution maps and modelling

Ecological niche modelling (ENM) was carried out in MAXENT v 3.3.3e [56] to predict suitable present and past potential distribution areas of *O*. *nasutus* based on climatic variables. Such modelling has been performed favourably compared to other analytical alternatives for presence-only data [56–59]. Information on the geographic distribution of the species was based on 51 occurrence records obtained from literature and collections (S2 Appendix). We verified the accuracy of the geo-referenced data in ArcGIS v 10.0. To avoid data clustering [60], we limited our database to a single record per km^2^. For modelling settings, the function ‘Auto features’ was selected, and the distributions were modelled through the ‘cross-validate’ parameter, applying a maximum number of iterations at 500. We verified model performance using the area under the ‘Receiver Operating Characteristic (ROC) Curve’ (AUC) calculated by MAXENT. Values between 0.7 and 0.9 indicated good discrimination [61].

Projections for the past climatic conditions were developed for three periods: LGM c. 21 ka; Last IinterGlacial (LIG) c. 120–140 ka; and mid-Holocene c. 6 ka. Current and past distributions were modelled using 19 bioclimatic data layers available from the WorldClim database (http://www.worldclim.org) at 30 arc-sec resolution (~1 km^2^), with the exception of the layers from the LGM period, which were available in 2.5 arc-min resolution (~5 km^2^). Climatic variables for the present study represented the average climate changes from 1950 to 2000. Projections for the LGM and mid-Holocene were derived from the CCSM4 [62] atmosphere-ocean general circulation models (AOGCM). All the analyses were performed in QuantumGIS 2.18 software.

## Results

### Intraspecific genetic variation and evolutionary patterns

We identified 73 variable sites in the mtDNA dataset resulting in 38 haplotypes and 32 variable sites in the *Fgb*-I7 locus resulting in 40 haplotypes (with one 2-bp indel) (Tables 1 and 2). Standard diversity indices (haplotype diversity, nucleotide diversity, mean number of pairwise differences) for both the markers are presented in Table 2.

**Table 2:** Geneticvariability of *O*. *nasutus* using mitochondrial (*Cytb*) and nuclear (*Fgb*-I7) markers.

Group		S	N_H_	Hd ± SD	π ± SD	k	Fu-Fs (P-value)	Tajima’s D (P-value)
*Cytb*:								
Northwest	4	12	4	1.0000 **±** 0.1768	0.00885 ± 0.00256	6.83	-0.12436 (0.2570)	0.44358 (0.7550)
Central	13	10	6	0.6282 **±** 0.1431	0.00229 ± 0.00093	1.76	-0.36009 (0.3760)	-1.80161 (0.0160)*
Eastern	18	8	7	0.7451 **±** 0.0790	0.00280 ± 0.00037	2.16	-0.24573 (0.4710)	-0.24280 (0.4240)
Steppes Plain	30	18	12	0.8874 **±** 0.0329	0.00379 ± 0.00047	2.92	-3.34189 (0.0670)	-1.22763 (0.1100)
Southern	13	11	8	0.9103 **±** 0.0559	0.00422 ± 0.00065	3.25	-1.96098 (0.1040)	-0.32877 (0.4000)
Taim Wetland	4	6	3	0.8333 **±** 0.2224	0.00410 ± 0.00172	3.16	0.81143 (0.5720)	-0.31446 (0.5510)
All	82	73	38	0.9624 **±** 0.0083	0.01190 ± 0.00066	9.18	-11.24103 (0.0070)**	-1.23865 (0.0720)
*Fgb*-I7:								
Northwest	3	8	5	0.9333 ± 0.1217	0.00640 ± 0.00123	3.66	-0.90493 (0.2050)	-0.06042 (0.4550)
Central	8	14	14	0.9500 ± 0.0364	0.00606 ± 0.00062	3.45	-4.51905 (0.0060)**	-1.22325 (0.1120)
Eastern	16	13	14	0.8407 ± 0.0557	0.00481 ± 0.00053	2.92	-5.23937 (0.0140)*	-0.90384 (0.1990)
Steppes Plain	29	11	14	0.7629 ± 0.0538	0.00293 ± 0.00052	2.00	-5.31470 (0.0160)*	-1.05889 (0.1440)
Southern	9	13	11	0.9216 ± 0.0417	0.00639 ± 0.00098	3.53	-3.67515 (0.0280)	-0.68862 (0.2940)
Taim Wetland	3	0	1	-	-	-	-	-
All	68	32	40	0.8778 ± 0.0225	0.00497 ± 0.00034	2.98	-26.44071(0.00)**	-1.93728 (0.0050)**

**Fig 2:**
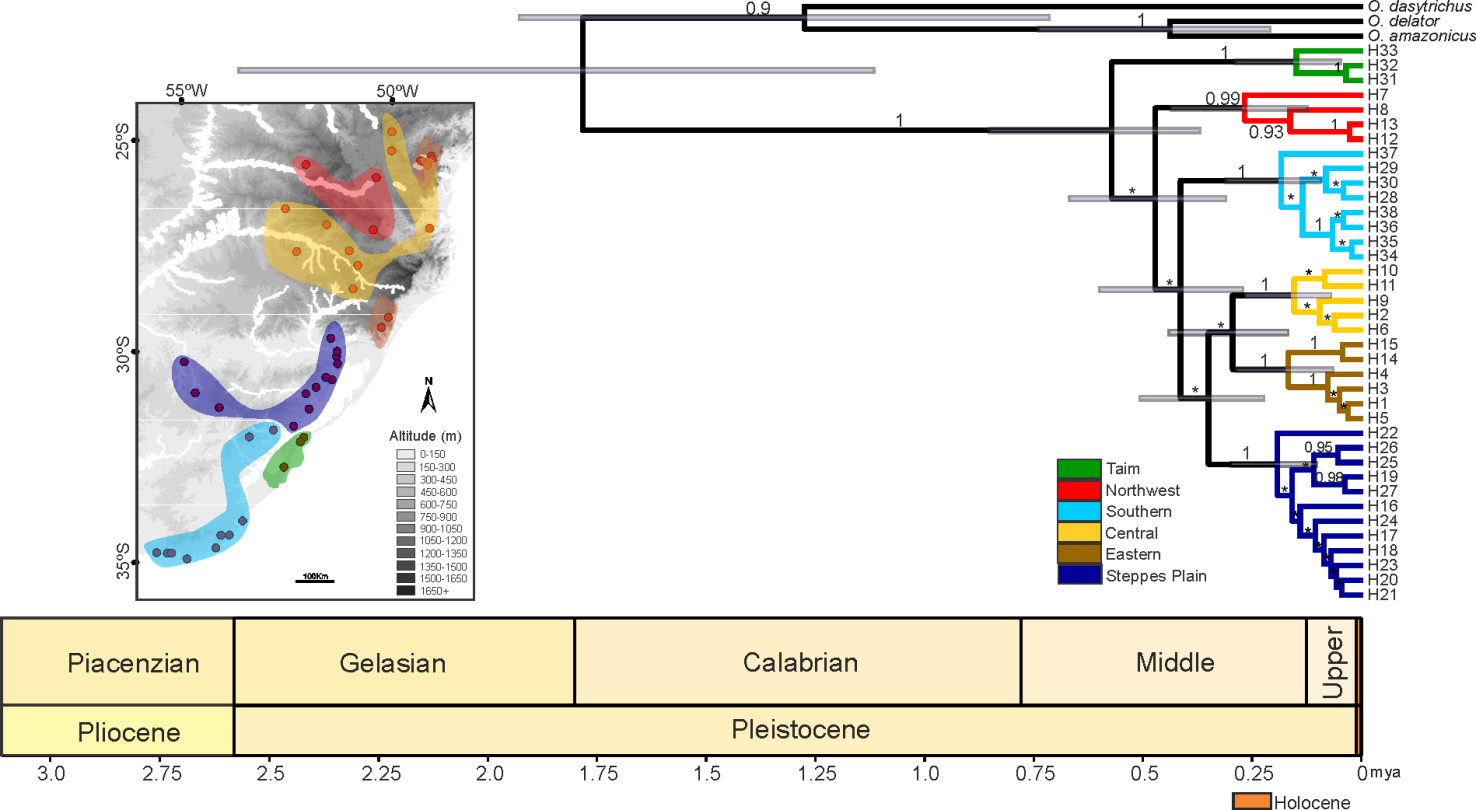
Bayesian consensus time-tree of *O*. *nasutus* based on 801 bp of the *Cytb* mtDNA sequences. Values above nodes correspond to posterior probabilities > 0.90. The 95% credible intervals for node ages are shown with transparent bars to denote the time of the most recent ancestor (TMRCA) to selected branches. Scale bars were plotted with a geological time scale using the strap package in R. The map depicted clades into a geographic context, highlighting distinct altitudes.

Groups are based on *Cytb* phylogenetic inferences (see Fig 2). Neutrality tests are indicated by Fu’ Fs and Tajima’s D.

N_ind_: Number of individuals sequenced, S: Number of segregating sites, N_H_: number of haplotypes; Hd: Haplotype diversity, **π**: Nucleotide diversity, SD: Standard deviation; k = Mean number of pairwise differences.

*P<0.02 or

**P<0.01 for Fu’s FS or Tajima’s D, respectively.

**Fig 3:**
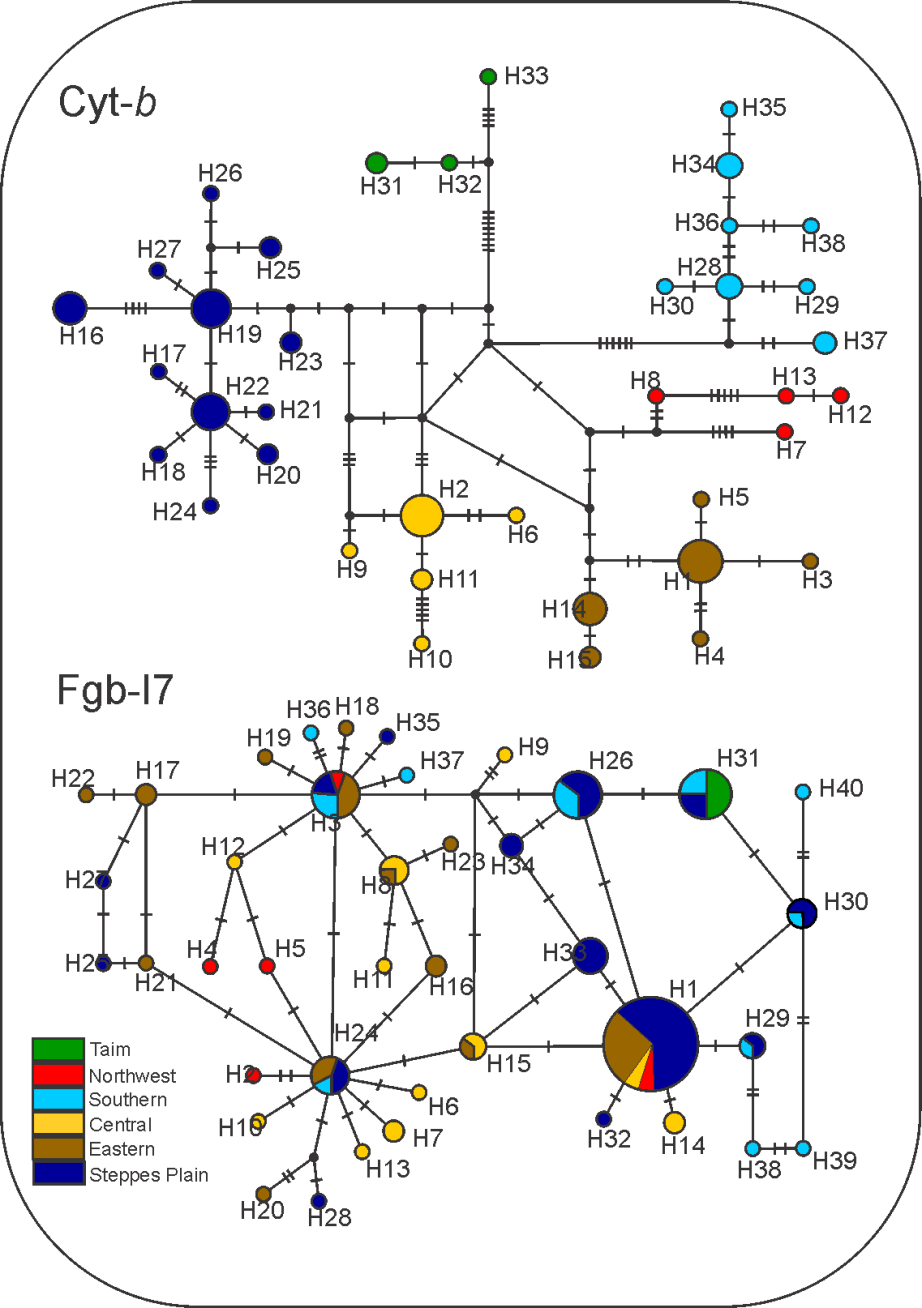
Evolutionary relationship of *O*. *nasutus* haplotypes. Median joining network based on the mitochondrial *Cytb* fragment and the nuclear *Fgb*-I7 locus. Color coding denotes the major mtDNA clades obtained in the Bayesian dated phylogeny (see Fig 2).

Bayesian consensus tree based on mtDNA dataset depicted six highly supported (BPP > 0.90) haplogroups corresponding to two distinct biomes: Northwest, Central, and Eastern clades are distributed along the Atlantic Forest biome, and Steppes Plain, Taim Wetland, and Southern clades assigned to the Pampas biome. However, the relationship between these groups was not entirely clear, considering that they did not cluster in major clades exclusive to each of the two biomes (Fig 2). The haplotype network based on the mtDNA also supported such tree structure. Nonetheless, despite the several mutational steps between haplogroups, the haplotype network showed several median vectors indicating non-sampled or extinct ancestral sequences (Fig 3). The two main widespread haplogroups were Central and Southern clades. The Central clade occurred throughout the domain of Atlantic Forest, mainly dispersed over the Araucaria forests and the mosaic of forest/highland grasslands (Fig 2). This clade was distributed across the entire extension of the Araucaria Forest coverage. The Southern clade was dispersed along the Pampas biome in Uruguay and the southernmost of Brazil, a region dominated by steppes or grassland. The other haplogroups were isolated and covered smaller areas when compared to Central or Southern haplogroups, except for Eastern clade with two isolated zones of occurrence. The most diversified haplogroup was the Steppes Plain, including 12 distinct haplotypes dispersed throughout the state of Rio Grande do Sul, Brazil (Table 1). Only Piraquara locality in Parana state, Brazil, comprised haplotypes from two distinct mtDNA clades (Fig 1). nDNA network revealed 36 low-frequency haplotypic variants, with a reticulate evolutionary relationship.

The majority of haplotypes differed by one substitution site. None of the six major clades in the mitochondrial tree was recovered for the *Fgb*-I7 sequences. However, nuclear haplotype H1 was present in almost all mtDNA clades (except Taim Wetland). All mtDNA clades showed the presence of unique alleles, but Taim Wetland group shared the haplotype 31 with Steppes Plain and Southern clades.

The genetic distances between the Bayesian clades ranged from 1% to 2.5% for mtDNA, whereas it was close to 0 for *Fgb*-I7 (S1 Table).

The most common ancestor for all clades of *O*. *nasutus* was estimated to the middle Pleistocene (0.5715 myr; 95% HPDs = 0.3657–0.8471 mya) (Fig 2, Table 3). The mtDNA haplotypes (n = 38) clustered into six lineages; individual clades diverged between 265.8 and 147.3 myr (HPDs = 0.438.8–0.046.7 myr). The ancestral haplotype could not be inferred for this marker due to the presence of several median vectors in the network. However, the BEAST-derived tree indicated H7 (Northwest clade) as the oldest clade. Tajima’s D and Fu’s Fs tests were negative and non-significant for both mtDNA and nDNA markers, indicating that *O*. *nasutus* might have experienced a recent population expansion (Table 2).

**Table 3:** Estimates of the time to the most recent common ancestor (TMRCA) for the nodes addressed in this study using unique haplotypes for each of the mitochondrial and overall clades for *O*. *nasutus*.

Clade	TMRCA (Ma)	95% HPD
Northwest	0.2658	0.1275–0.4388
Central	0.1529	0.0691–0.2675
Eastern	0.1658	0.0689–0.2894
Steppes Plain	0.1911	0.1057–0.3017
Southern	0.1822	0.0905–0.3087
Taim Wetland	0.1473	0.0467–0.2902
All	0.5715	0.3657–0.8471

Only Fu’s Fs test showed significant P values for mtDNA (P < 0.01). Considering the mtDNA, only the Central haplogroup depicted significant P values in Tajima’s D test (-1.80161, P = 0.0160), indicating an undergoing demographic expansion or mutational selection (too many segregating sites/too few pairwise differences). On the other hand, Northwest haplogroup showed positive values for Tajima’s D test (0.44358, P = 0.7550), which could be an indicator for a contraction (too few segregating sites/too many pairwise differences). Central, Eastern, and Steppes Plain clades showed significant P values for Fu’s Fs tests performed on nDNA marker, as expected from a recent population expansion. This indicated recent demographic expansion or departure from the null hypothesis of selective neutrality and population equilibrium. Nonetheless, a unique haplotype for nDNA was found in Taim Wetland mtDNA clade, which did not allow neutrality tests to be performed. This clade also showed positive values for the Fu’s Fs test for mtDNA, thereby suggesting recent population bottleneck for this lineage (Table 2).

**Fig 4:**
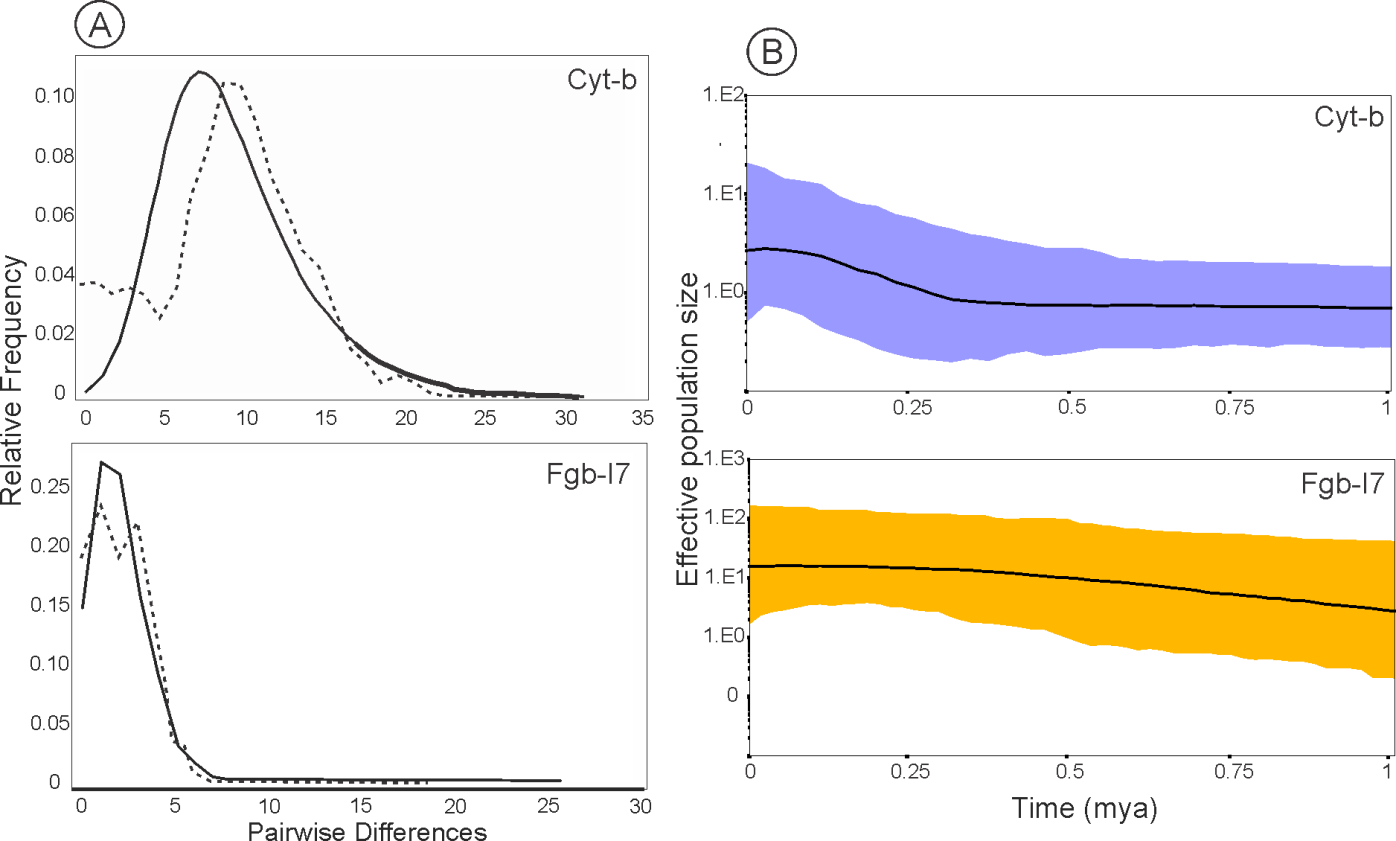
Demographic history of *O*. *nasutus* with signatures for population expansion. A, mismatch distributions of pairwise differences of *Cytb* and *Fgb*-I7 haplotypes obtained under a model allowing expansion. B, Bayesian Skyline Plot for *Cytb* and *Fgb*-I7 datasets. Bold lines indicate the median of effective population size through time and the coloured lines represent the 95% highest posterior densities over the median estimates along the coalescent history of the species.

The results of the mismatch distribution for mtDNA and nDNA analysis was approximately unimodal (Fig 4). Non-significant SSD statistic (SSD = 0.00112542, P = 0.944) and raggedness index value (r: 0.0032, P = 0.9770) under the spatial expansion models failed to reject the spatial expansion model. Similar patterns suggested demographic expansion for nDNA (SSD = 0.00322295, P = 0.570; r: 0.01631205, P = 0.780). Strong evidence of demographic expansion came from the BSP. The results showed different patterns between the mitochondrial and the nuclear datasets. For the mtDNA, a constant population size was noted over the last 0.375–1 mya after a long phase of demographic stability; the population then appeared to have experienced an accelerated demographic expansion phase approximately 0.030–0.300 myr, followed by a decrease in population size after 0.030 myr. For the nDNA marker, results showed a slow growth in the effective population size across time (0.400–1.2 mya), demographic stability for 30–400 myr, with smooth decrease posteriorly (Fig 4).

### Skull morphometric variation

**Fig 5:**
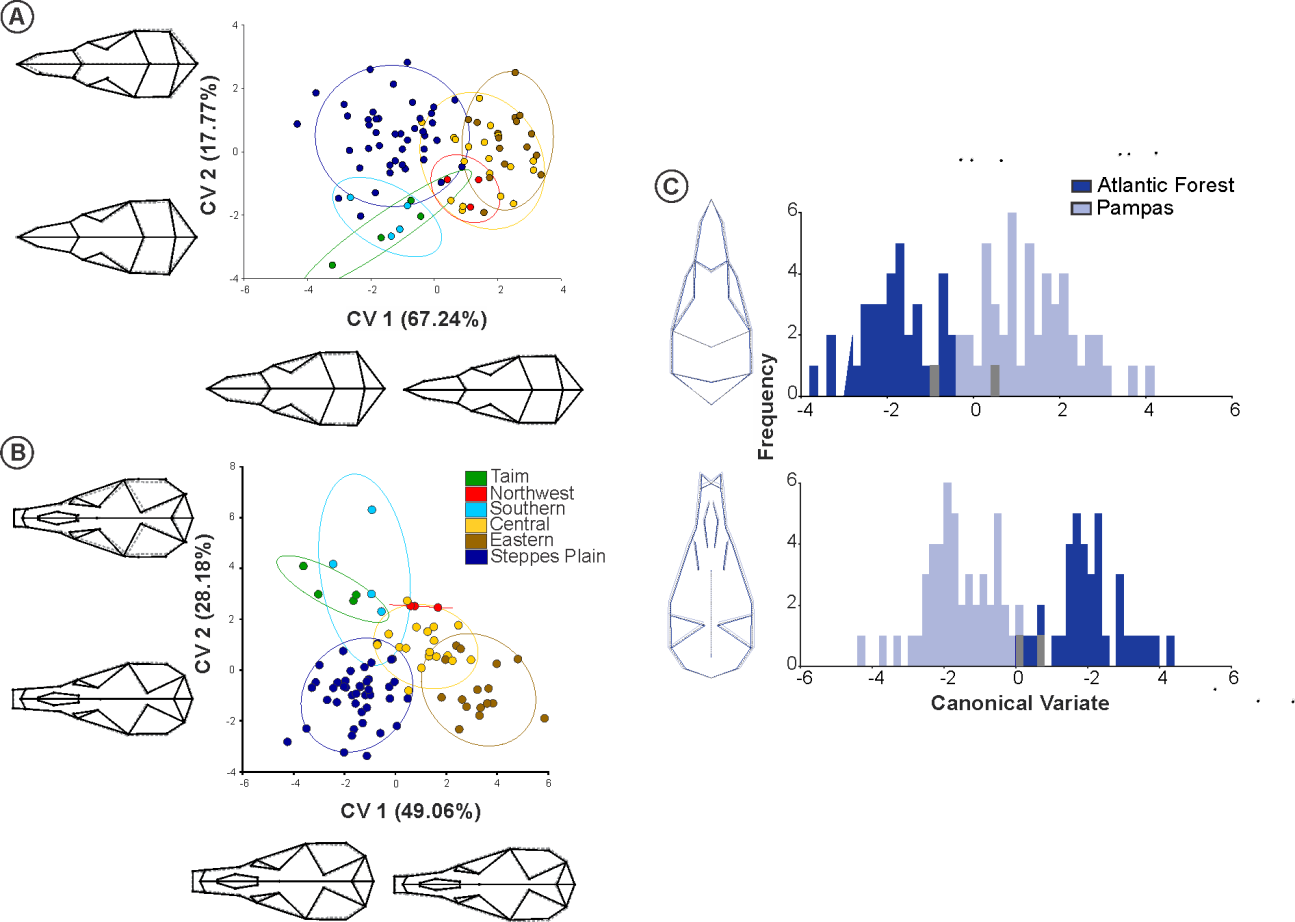
**Scatter plot of the first Canonical Variate axis of the *O*. *nasutus* skull at the dorsal and ventral views, with groups following the genetic haplogroups (A, B), and the physiognomies (C).** Changes in the shape for each axis are given; solid lines indicate positive scores and dashed lines indicate negative ones.

The occupation of different physiognomies, or environmental groups (Atlantic Forest or Pampas) explained 17.3% of the variation in the dorsal view (Wilk’s Λ = 0.271, p < 0.001), 17.9% in the ventral view (Wilk’s Λ = 0.23, p < 0.001) (Fig 5), and 15.4% in the lateral view (Wilk’s Λ = 0.31, p < 0.001) of the *O*. *nasutus* skull (S3 Fig). Genetic haplogroups indicated 24.7% of the variation in the dorsal view (Wilk’s Λ = 0.084, p < 0.001), 26.3% in the ventral view (Wilk’s Λ = 0.014, p < 0.001) (Fig 5), and 23.5% in the lateral view (Wilk’s Λ = 0.021, p < 0.001) of the *O*. *nasutus* skull (S3 Fig). The percentage of correct classification among the haplogroups were: 62.92% for the dorsal view, 65.16% for the ventral view, and 55.05% for the lateral view. Among the environmental groups, the percentages of correct classification were: 93.25% for the dorsal view, 82.02% for the ventral view, and 80.89% for the lateral view. The percentages of correct classification were similar between the genetic and the environmental haplogroups, considering the total number of groups in each one (i.e. six haplogroups would result in ~16% of the correct classification by chance, while with two groups this percentage will rise to 50%; deviations from the random classification are similar between the predictors). Detailed results of discriminant analyses can be found in the S2 and S3 Tables.

### Ecological niche modelling and ancestral area reconstruction

**Fig 6:**
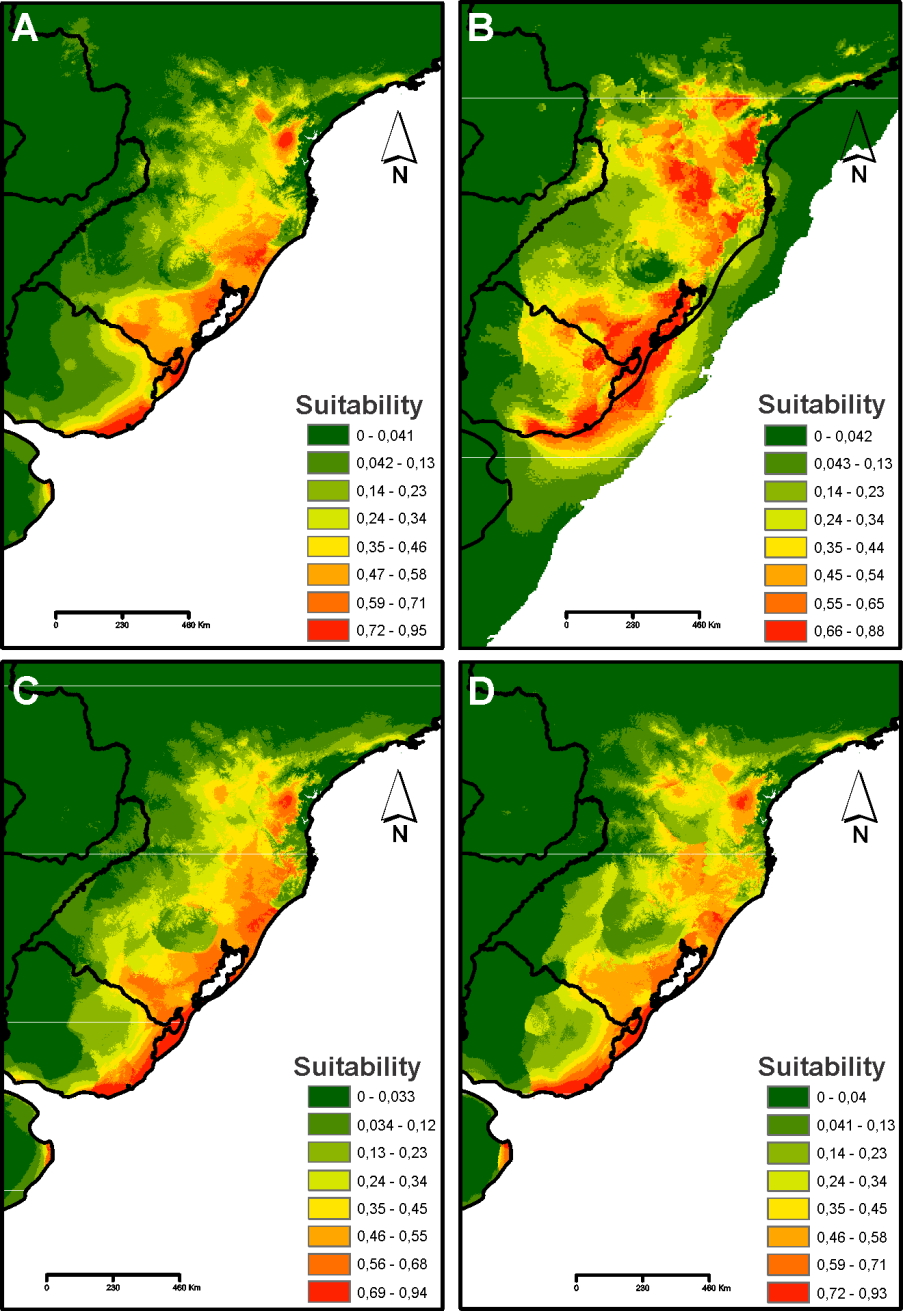
Predictive distribution models for *O*. *nasutus*. Warmer colours depict areas of higher predicted suitability. (A) Last Interglacial “LIG” ~120–140 kya. (B) Last Glacial Maximum “LGM” ~21 kya. (C) Mid-Holocene ~6 kya. (D) Current conditions. Maps were obtained from “OpenStreetMap contributors” (available at: www.openstreetmap.org; Open Street Map is made available under the Open Database License: http://opendatacommons.org/licenses/odbl/1.0/. Any rights in individual contents of the database are licensed under the Database Contents License: http://opendatacommons.org/licenses/dbcl/1.0/), and edited with QGis 2.18 software. The images were also edited using Corel Draw graphics Suite (X5).

Relatively high AUC values showed an excellent predictive power of the ENMs (current, AUC: 0.952; MID-HOL, mean AUC: 0.952; LGM, mean AUC: 0.952; LIG, AUC value: 0.958). The ENM results (Fig 6) and the potential LGM distributions were more widespread. On the other hand, the potential distribution of MID-HOL was reduced, which showed more similarities with the current conditions. The potential distributions of the LIG were the most restricted of all the ENMs. Suitable habitats in the LGM showed a great expansion in relation to the LIG. However, in all the models tested, areas of low suitability present between the Pampas and Atlantic Forest domains were identified, as well as in the western part of the territory of Uruguay. In addition, the coastal areas did not have suitable conditions during the LGM.

**Fig 7:**
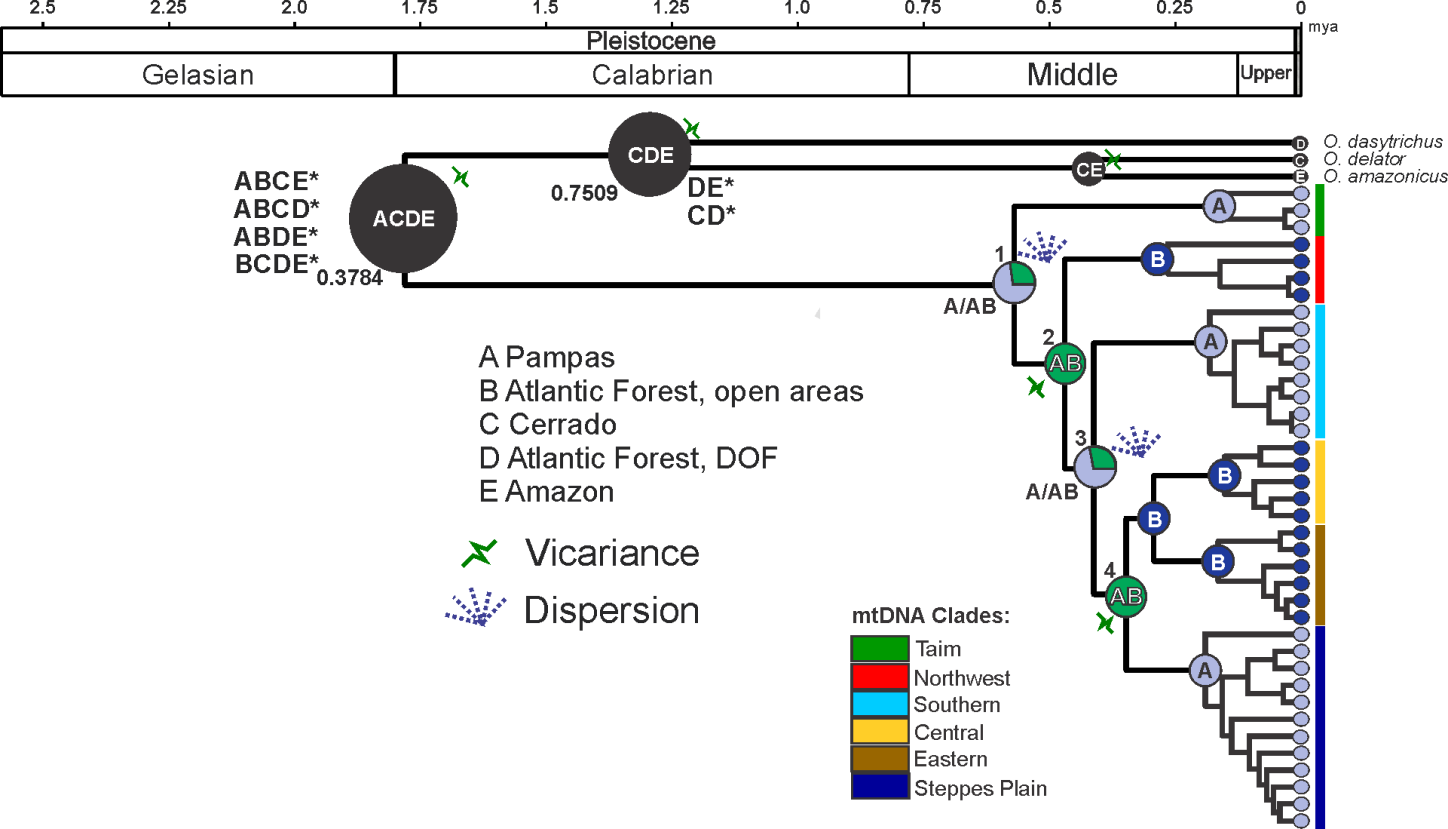
BEAST–derived phylogenetic relationships of 38 mtDNA haplotypes, divergence dating, and ancestral area reconstruction of *O*. *nasutus* clades. Larger pie diagrams on the nodes indicate the ancestral distributions inferred by S-DIVA methods using RASP. Asterisk represents the occurrence of less frequent inferences. Symbols indicate dispersion or vicariance. The clades are denoted according to Bayesian clades. Area codes: A-Pampas; B- Southern Atlantic Forest; C-Cerrado; D-Ombrophylous Dense Forest; E-Amazon.

According to S-DIVA analysis (Fig 7), Pampas ecoregion (A/AB, node 1) was the most likely region for the origin of the dispersal of *O*. *nasutus*. Dispersal events probably progressed from Pampas domain (A) to areas that are currently translocated into the Atlantic Forest domains (B) and also ranging to other regions of the Pampas. Vicariance signals, detected on node 2 (AB), indicated that vicariance events occurred between the Northwest clade and the other haplogroups that had dispersed between the Pampas and the Atlantic Forest. A new dispersion event was detected on node 3 (A/AB), scattering the haplogroups (Southern, Steppes Plain, Central, and Eastern) along the Pampas and the current Atlantic Forest areas. Finally, on node 4, a new vicariance was verified between the haplogroups that were present on the Atlantic Forest areas (Central and Eastern) and the Pampas (Steppes Plain).

## Discussion

### Genetic and morphological variation in the Pampas and Atlantic Forest

The absence of *Cytb* haplotypes that was common to Atlantic Forest and Pampas groups was intriguing. However, the scenario was intricate, as a lack of further structuring between the two biomes in the Bayesian and median-joining network analysis was evident. This pattern indicated a possible isolation following the historical scenario of gene flow, as depicted by the *Fgb*-I7 network. Patterns of skull form were also roughly in agreement with the molecular findings. The differentiation between the physiognomies of *O*. *nasutus* populations were evident in the classification after the cross-validation. Subtle differences in the skull shape of *O*. *nasutus* populations were also depicted among the environments. Such skull differences might be related to genetic grouping or with environmental factors, exerting some form of local selection and/or guiding phenotypic plasticity in each physiognomy [8]. Similarly, studies on haplogroups of sympatric akodonts (e.g., *Deltamys kempi* [63, 64] and *Scapteromys* spp. [65, 66]). were also divergent in the geometric or linear morphometric analysis of the skull.

A conspicuous geomorphological feature of the connection among the southern Atlantic Forest and Pampas landscapes is the escarpment of Serra Geral (Meridional Plateau), which might have acted as a long-term barrier to gene flow between the Pampas and the current Atlantic Forest open areas at the eastern point of contact of these two biomes. Thus, the altitudinal gradient formed might have influenced certain level of differentiation, thereby rendering a group of lineages dispersing across the highlands of the Meridional Plateau, and others through the sedimentary lowlands of the Paraná basin, and later spreading along the coastal plain. Mountain ranges and ridges have been considered as topographic elements associated with speciation processes in akodontine rodents [67–71]. Since the differentiation in *O*. *nasutus* populations is recent (time-tree revealed that the diversification probably began in the Middle Pleistocene) and the divergence process seems incomplete, it can be considered at the population-level and not at species-level yet.

The population structure of *O*. *nasutus* was quite complex; therefore, it is possible that the recent expansion of forest formations of the South Atlantic Forest, such as the Araucaria Forest and the seasonally dry Forest lato sensu [21], along with the escarpment of Serra Geral have created a new geographical barrier that separated the Atlantic Forest and Pampas lineages. Hence, divergence in the mtDNA clades suggested that the lineages have persisted in isolation across time, characterizing an ‘grassland refuge’ pattern. Accordingly, the species adapted to dry and/or cold environments and restricted to small areas surrounded by unsuitable habitats can persist through interglacial microrefugium [72].

### Phylogeographic patterns

The phylogenetic tree revealed a more complex evolutionary history in the open areas of Pampas and Atlantic Forest instead of simple reciprocally monophyletic groups. Six major mtDNA clades were observed, which fall within the range of intraspecific divergence described for the Akodontini tribe [63, 64, 71, 73]. The mtDNA haplotype network analysis in this study was congruent with such tree structure, representing the six haplogroups separated by several mutational steps, and inserted in specific ecoregions. However, median joining vectors present in the network did not rule out the hypothesis of non-sampled or possibly extinct haplotypes. Although differentiation was less evident, patterns found in nDNA haplotypes provided indication of a demographic expansion event. Similarly, results of mtDNA analysis also suggested demographic growth. In addition, intraspecific *Fgb*-I7 variability was conspicuously lower than *Cytb* variability, as reported in other studies [43, 63,74]. Molecular markers such as mtDNA and nDNA generally show different phylogeographic patterns for the same biogeographical history owing to differentiation in effective population sizes, recombination, and mutation rates [75, 76].

The estimates of genetic distances for the mtDNA clades were moderately high (up to 2%). This might be the result of an ongoing process of isolation, and because mtDNA accumulates substitutions 5–10 times faster than the single-copy nuclear DNA [75]. The highest distances were observed among Taim Wetland versus other clades and between Southern and Northwest clades; however, none of these genetic distances were recovered for the nuclear locus (divergence close to zero). The highest genetic distance observed for these clades (Taim Wetland, Northwest, and South) might relate to the ancestral condition of these groups, as depicted by BI and mtDNA network, along with the fact that they are geographically distant from each other. According to the S-DIVA analysis, tandem dispersion events followed by vicariance might have probably influenced the phylogenetic pattern of mtDNA observed in the study.

The Northwest clade was the northernmost lineage; so, the effects of the dynamics of forest formations/open areas might have reached earlier in this group, making it more suitable to environmental pressure. Positive values for the Tajima’s D test (0.44358, P = 0.7550) for this clade (from mtDNA) and other negative but non-significant values (for both markers), indicated a possible contraction effect on this lineage. The divergence between the Steppes Plain and Central and Eastern clades dates back to 0.350 myr (0.220–0.505 myr). Moreover, some forest formation similar to Seasonal Forest, which at present is adjacent to the escarpment of the Serra Geral might have acted concomitantly. This hypothesis was supported by the results of the ENM that indicated an area of very low suitability between the zone of contact of these two ecoregions. This is consistent with the escarpment of Serra Geral, which might have corroborated mainly due to the vicariance between the Steppes Plain and Central and Eastern clades, as detected by S-DIVA analyses. Similarly, a strong phylogenetic break in this region was evident for other akodonts recently described, such as *Scapteromys meridionalis* [71] and *Deltamys araucaria* [64]. An evident pattern is the genetic divergence within the Southern clade in the Pampas ecoregion (Uruguay). In this study, ENM supported up to a limited dispersion, indicating a possible historical barrier acting in this region. In this case, the Rio Negro could be possibly acting as a barrier for Southern clade, wherein areas of low suitability were identified in all the prediction models. Southern clade was one of the most spread throughout the Pampas ecoregion. Although neutrality test indicated expansion, Southern clade was the only clade where suitable areas were identified mainly in the coastal regions and continental shelf, according to the LGM niche models.

A remarkable phylogeographic aspect was the genetic divergence of the Taim Wetland clade. The mtDNA dataset revealed a statistically supported phylogenetic break between the Taim Wetland clade and other clades, indicating a possible historical barrier acting in this region. This lineage originated about 0.147 myr (95% HPD = 0.047–0.290 myr) and was the first to have dispersed in the S-DIVA analysis. This clade was inserted on the plains of southernmost Brazil (RS), where climatic oscillations occurred during the middle Pleistocene and Holocene, resulting in marine transgressions and regressions of relative sea level that shaped the South Atlantic Coastal Plain (SACP). The formation of the SACP is related to the sedimentary processes associated with the sea transgressive events known as ‘Barriers’, which occurred in the middle Pleistocene with the formation of Barrier I (~ 0.400 myr), Barrier II (~ 0.325 myr), Barrier III (~ 0.120 myr), and Holocene, when Barrier IV (~0.005 myr) was established [77]. Possibly, this clade accessed this region after the formation of barriers II and III, between 0.325 and 0.120 myr, respectively. The geological evolution of the “Barriers” also formed the Patos-Mirim complex, the largest lagoon-barrier system in South America [77]. In addition, three paleochannels related to the Jacuí, Camaquã, and Jaguarão rivers were identified in the middle and southern coastal zone of RS [78]. These paleochannels were associated with the large persistent hydrographic elements such as Mirim Lagoon, São Gonçalo Channel, and the Patos Lagoon estuarine channel, which might have possibly contributed to the isolation of this lineage in the southernmost domains of the SACP. Similarly, recent studies show a strong phylogenetic break in the same region for *Scapteromys tumidus* [65] and *Deltamys kempi* [64]. Thus, major elements of Patos-Mirim lagunar complex might constitute a geographical barrier for the historical gene flow.

### Climate change and landscape

Overall, substantial demographic changes associated with Pleistocene climatic oscillations were observed in more than half of the biota investigated in South America. Considering only the taxa to be associated with open vegetation, a total of 68% of the studied species experienced population expansion during the glacial periods [6]. The historical biogeographic approach presented herein appeared to corroborate this pattern detected for the open areas-dwelling species.

All divergence times within *O*. *nasutus* populations estimated by BEAST occurred during the Middle Pleistocene, wherein a minimum of eight glaciations occurred in the Middle–Late Pliocene in the southern Andes of South America [79]. However, the greatest glaciations occurred during the early Pleistocene, such as the Great Patagonian Glaciations, between 1.16 and 1.01 mya, when the glaciers advanced up to 200 km east of the Andes mountains, and stretching along the Pacific and south of Atlantic coast [79,80]. After the Great Patagonian Glaciations, 13 minor glacial and interglacial periods were recorded for this region in the Early-Middle Pleistocene that reached a maximum around 25,000 and 16,000 years BP (see [79]).

Although the emergence of *O*. *nasutus* occurred in the earlier periods (ca. 500 kyr), the patterns showed by the present and past niche models depicted expansion of high probability areas during the LGM (~21,000 years BP), favouring its distribution. Probably, a pattern similar to LGM might have occurred during the Great Patagonian Glaciations, where the effects of glaciations might have increased the open areas and retracted the forest formations. The areas of Southern Atlantic Forest were dominated by grasslands between 42,000–10,000 years BP; the period comprising the LGM. Forest elements were restricted to sites in deep river valleys and in coastal lowlands, indicating a cold and dry climate [21]. In general, grasslands adapted to cold conditions prevailed in the southern and southeastern Brazil until around 11,500 years BP [81]. Since *O*. *nasutus* is intrinsically related to open areas and is abundantly found in these habitats [17–19], cyclic events of the dynamic expansion and retraction of open areas and forests that occurred during glacial and interglacial periods have probably caused species dispersal and historical distribution. Such expansions of open areas promoted by glaciations made the environment more suitable for establishment of new areas, which was detected by S-DIVA analysis. It was interesting to reveal that no suitable areas in the coastal zone were increased during LGM according to the niche model. Palynological studies on the coasts of the states of Santa Catarina and Paraná indicated that fields were abundant in this region and also on the exposed continental shelf during the LGM period, whereas tropical tree species were practically absent [23]. The continental shelf during LGM presented a dry climate, hampering the colonization by any species adapted to humid/wet habitats. Areas of moderate suitability were found in the coastal region of southern Brazil and Uruguay during the LIG, Middle Holocene, and current models, with a reduction of suitability in the inland areas. Thus, the coastal regions in the Pampas ecoregion might have served as a refuge for *O*. *nasutus* during the interglacial periods.

*O*. *nasutus* is known to inhabit the subtropical grassland in wet or flooded seasons [17]. Accordingly, the species is adapted to humid environments, being found in bunch grass and in tall grass near the streams and rivers [82], sandbanks near wet lands across coastal semi-fossorial habit [19], and coastal vegetation in the South Atlantic Coastal Plain [83]. Thus, in addition to grassland, *O*. *nasutus* might have followed river routes in search of humid environments. Such habitat specialization might reflect the dispersion ability to more extensive regions during the glacial periods.

In this context, we suggest that the two major rivers in Rio Grande do Sul state (the Jacuí and Ibicuí) might have acted (and still be acting) as river barrier for the Steppes Plain clade. The haplotypes of this clade are distributed across the western edge of the Patos Lagoon and the interior of the plains of southern Brazil. Therefore, ENM suggested that these areas might be unsuitable for the restriction caused mainly by the river Jacuí. Others rivers could have affected the limits of dispersion in *O*. *nasutus*. Rivers Uruguay and Paraná also might have acted in limiting the dispersion during the glacial periods. The paleoecological niche modelling suggested that at LGM, the River Uruguay limited the dispersion for Steppes Plain and Southern clades in Uruguay and Southern Brazil to reach new areas to the west. Similarly, the River Paraná limited the dispersion for Northwest and Central Clade in highlands. Finally, Paraná, and Paranapanema rivers might have acted as physical barriers in the dispersion to the north, mainly for the Northwest clade, due to their amplitude and water volume. These inferences were based on the ENM results, wherein areas of low suitability were verified in all the prediction models.

## Supporting information

S1 Fig Bayesian consensus time-tree based on the *Fgb*-I7 sequences. Values above nodes correspond to posterior probabilities > 0.90.
(TIF)

S2 Fig Position of the landmarks (circles) digitized on the dorsal, ventral, and lateral views of the *O*. *nasutus* skull. A description of each landmark is presented in S1 Appendix.
(TIF)

S3 Fig Scatter plot of the first Canonical Variate axis for the skull of *O*. *nasutus* in the lateral view, with groups following the genetic haplogroups (A, B) and the physiognomies (C). Changes in the shape for each axis are given. Solid lines indicate positive scores and dashed lines indicate negative ones.
(TIF)

S1 Appendix Definition of the landmarks positioned at the three skull views of *O*. *nasutus* analysed specimens (see. S2 Fig). (DOCX)

S2 Appendix Additional localities retrieved from the literature for the spatial distribution modelling analysis. (DOCX)

S1 Table Genetic divergence (using *p-*distance) between pairs of *Cytb* haplotypes recovered from different clades of *O*. *nasutus* determined by the Bayesian phylogeny. (DOCX)

S2 Table Percentage of correct classification by discriminant analysis, using Jackknife cross-validation, for the dorsal, ventral, and lateral views of the *O*. *nasutus* skull for the mtDNA clades. (DOCX)

S3 Table Percentage of correct classification by discriminant analysis, using Jackknife Cross-validation, for the dorsal, ventral, and lateral views of the *O*. *nasutus* skull for the two ecoregions. (DOCX)

## References

[1] Lisiecki LE, Raymo ME. Plio-Pleistocene climate evolution: Trends and transitions in glacial cycle dynamics. Quaternary Sci Rev. 2007; 26, 56–69. doi: 10.1016/j.quascirev.2006.09.005

[2] Hewitt GM. Some genetic consequences of ice ages, and their role in divergence and speciation. Biol. J. Linn. Soc. 1996; 58: 247–276.

[3] Hewitt G. The genetic legacy of the Quaternary ice ages. Nature. 2000; 405: 907–913. doi: 10.1038/35016000 10879524 10.1038/35016000

[4] Hewitt G. Genetic consequences of climatic oscillations in the Quaternary. Philos. Trans. R. Soc. Lond. B: Biol. Sci. 2004; 359: 183–195. doi: 10.1098/rstb.2003.1388 15101575 10.1098/rstb.2003.1388 PMC1693318

[5] Avise JC. Phylogeography: the history and formation of species Cambridge, Harvard University Press; 2000.

[6] Turchetto-Zolet AC, Pinheiro F, Salgueiro F, Palma-Silva C. Phylogeographical patterns shed light on evolutionary process in South America. Mol Ecol. 2013; 22: 1193–1213. doi: 10.1111/mec.12164 23279129 10.1111/mec.12164

[7] Carnaval AC, Moritz C. Historical climate modelling predicts patterns of current biodiversity in the Brazilian Atlantic forest. J Biogeogr. 2008; 35: 1187–1201. doi: 10.1111/j.1365-2699.2007.01870.x

[8] Maestri R, Fornel R, Gonçalves GL, Geise L, Freitas TRO, Carnaval AC. Predictors of intraspecific morphological variability in a tropical hotspot: comparing the influence of random and non-random factors. J Biogeogr. 2016; 43: 2160–2172. doi: 10.1111/jbi.12815

[9] Bilenca DN, Miñarro FO. Identificación de áreas valiosas de pastizal en las pampas y campos de Argentina, Uruguay y sur de Brasil. Buenos Aires: Fundación Vida Silvestre Argentina 323 p. 2004

[10] Behling H. South and southeast Brazilian grasslands during Late Quaternary times: a synthesis. Palaeogeogr Palaeocl. 2002; 177: 19–27. doi: 10.1016/S0031-0182(01)00349-2

[11] Mapelli F, Mora MS, Mirol PM, Kittlein M. Effects of Quaternary climatic changes on the phylogeography and historical demography of the subterranean rodent *Ctenomys porteousi*.J Zool. 2012; 286: 48–57. doi: 10.1111/j.1469-7998.2011.00849.x

[12] Mora MS, Cutrera AP, Lessa EP, Vassallo AI, D’Anatro A, Mapelli FJ. Phylogeography and population genetic structure of the Talas tuco-tuco (*Ctenomys talarum*): integrating demographic and habitat histories. J Mammal. 2013; 94: 459–476. doi: 10.1644/11-MAMM-A-242.1

[13] Fregonezi JN, Turchetto C, Bonatto SL, Freitas LB. Biogeographical history and diversification of *Petunia* and *Calibrachoa* (Solanaceae) in the Neotropical Pampas grassland. Bot J Linn Soc. 2013; 171: 140–153. doi: 10.1111/j.1095-8339.2012.01292.x

[14] Felappi JF, Vieira RC, Fagundes NJR, Verrastro LV. So Far Away, Yet So Close: Strong Genetic Structure in *Homonota uruguayensis* (Squamata, Phyllodactylidae), a Species with Restricted Geographic Distribution in the Brazilian and Uruguayan Pampas. PLoS One. 2015; 10(2): e0118162 doi: 10.1371/journal.pone.0118162 25692471 10.1371/journal.pone.0118162 PMC4334718

[15] Cristiano MP, Cardoso DC, Fernandes-Salomão TM, Heinze J. Integrating Paleodistribution Models and Phylogeography in the Grass-Cutting Ant *D1* (Hymenoptera: Formicidae) in Southern Lowlands of South America. PLoS One. 2016; 11(1): e0146734 doi: 10.1371/journal.pone.0146734 26734939 10.1371/journal.pone.0146734 PMC4703384

[16] Bonvicino CR, Oliveira JA, D’Andrea PS. Guia dos Roedores do Brasil, com chaves para gêneros baseadas em caracteres externos. Rio de Janeiro: Centro Pan-Americano de Febre Aftosa—OPAS/OMS; 2008.

[17] Oliveira JA, Gonçalves PR. Suborder Myomorpha: Family Cricetidae: Subfamily Sigmodontinae. Genus *Oxymycterus*; pp. 247–268 in: Patton J.L., Pardiñas U.F.J. and D’Elía G. (eds.). Mammals of South America 2: Rodents. Chicago: University of Chicago Press; 2015.

[18] Paise G, Vieira EM. Daily activity of a Neotropical Rodent (*Oxymycterus nasutus*): seasonal changes and influence of Environmental factors. J Mammal. 2006; 87 (4): 733–739. doi: 10.1644/05-MAMM-A-158R5.1

[19] González EM.Guía de campo de los mamíferos de Uruguay Introducción al estudio de los mamíferos. Montevideo: Vida Silvestre 339p, 2001.

[20] Behling H, Bauermann SG, Neves PCP. Holocene environmental changes in the São Francisco de Paula region, southern Brazil. J S Am Earth Sci. 2001; 14: 631–639. doi: 10.1016/S0895-9811(01)00040-2

[21] Overbeck GE, Muller SC, Fidelis A, Pfadenhauer J, Pillar VD, Blanco CC, et al Brazil's neglected biome: The South Brazilian Campos. Perspect Plant Ecol. 2007; 9: 101–116. doi: 10.1016/j.ppees.2007.07.005

[22] Behling H, Pillar VD. Late Quaternary vegetation, biodiversity and fire dynamics on the southern Brazilian highland and their implication for conservation and management of modern Araucaria forest and grassland ecosystems. Philos T R Soc B. 2007; 362: 243–251. doi: 10.1098/rstb.2006.1984 10.1098/rstb.2006.1984 PMC2311428 17255033

[23] Behling H, Negrelle RRB. Tropical Rain Forest and Climate Dynamics of the Atlantic Lowland, Southern Brazil, during the Late Quaternary. Quaternary Res. 2001; 56:3, 383–389. doi: 10.1006/qres.2001.2264

[24] Smith MF, Patton JL. The diversification of South American murid rodents: evidence from mitochondrial DNA sequence data for the akodontine tribe. Biol J Linn Soc. 1993; 50: 149–177. doi: 10.1006/bijl.1993.1052

[25] Matocq MD, Shurtliff QR, Feldman CR. Phylogenetics of the woodrat genus *Neotoma* (Rodentia: Muridae): implications for the evolution of phenotypic variation in male external genitalia. Mol Phylogenet Evol. 2007; 42:637–652. doi: 10.1016/j.ympev.2006.08.011 17208019 10.1016/j.ympev.2006.08.011

[26] Katoh K, Standley DM.MAFFT Multiple Sequence Alignment Software Version 7: Improvements in Performance and Usability. Mol Biol Evol. 2013; 30: 772–780. doi: 10.1093/molbev/mst010 23329690 10.1093/molbev/mst010 PMC3603318

[27] Stephens M, Smith NJ, Donnelly P. A new statistical method for haplotype reconstruction from population data. Am. J. Hum. Genet. 2001; 68: 978–989. doi: 10.1086/319501 11254454 10.1086/319501 PMC1275651

[28] Stephens M, Donnelly P. A comparison of Bayesian methods for haplotype reconstruction from population genotype data. Am J Hum Genet. 2003; 73:1162–1169. doi: 10.1086/379378 14574645 10.1086/379378 PMC1180495

[29] Librado P, Rozas J. DnaSP v5: A software for comprehensive analysis of DNA polymorphism data. Bioinformatics. 2009; 25: 1451–1452. doi: 10.1093/bioinformatics/btp187 19346325 10.1093/bioinformatics/btp187

[30] Garrick RC, Sunnucks P, Dyer RJ. Nuclear gene phylogeography using PHASE: dealing with unresolved genotypes, lost alleles, and systematic bias in parameter estimation. BMC Evol. Biol. 2010; 10: 118 doi: 10.1186/1471-2148-10-118 20429950 10.1186/1471-2148-10-118 PMC2880299

[31] Darriba D, Taboada GL, Doallo R, Posada D. jModelTest 2: more models, new heuristics and parallel computing. Nat Methods. 2012; 30: 772 doi: 10.1038/nmeth.2109 10.1038/nmeth.2109 PMC4594756 22847109

[32] Drummond AJ, Suchard MA, Xie D, Rambaut A. Bayesian phylogenetics with BEAUti and the BEAST 1.7. Mol Biol Evol. 2012; 29:1969–73. doi: 10.1093/molbev/mss075 22367748 10.1093/molbev/mss075 PMC3408070

[33] Rambaut A, Drummond AJ. Tracer version 1.5 [computer program]. http://beast.bio.ed.ac.uk. 2009.

[34] Parada A D’Elía G, Palma R. The influence of ecological and geographical context in the radiation of Neotropical sigmodontine rodents. BMC Evol Biol. 2015; 15:172 doi: 10.1186/s12862-015-0440-z 26307442 10.1186/s12862-015-0440-z PMC4549906

[35] Hoffmann FG, Lessa EP, Smith MF. Systematics of *Oxymycterus* with description of a new species from Uruguay. J Mammal. 2002; 83: 408–420.

[36] Alfaro ME, Zoller S, Lutzoni F. Bayes or bootstrap? A simulation study comparing the performance of Bayesian Markov chain Monte Carlo sampling and bootstrapping in assessing phylogenetic confidence. Mol Biol Evol. 2003; 20: 255–266. doi: 10.1093/molbev/msg028 12598693 10.1093/molbev/msg028

[37] Bandelt HJ, Forster P, Röhl A. Median-joining networks for inferring intraspecific phylogenies. Mol. Biol. Evol. 1999; 16:37–48. doi: 10.1093/oxfordjournals.molbev.a026036 10331250 10.1093/oxfordjournals.molbev.a026036

[38] Kumar S, Stecher G, Tamura K. MEGA7: Molecular Evolutionary Genetics Analysis version 7.0 for bigger datasets. Mol Biol Evol. 2016; 33: 1870–1874. doi: 10.1093/molbev/msw054 27004904 10.1093/molbev/msw054 PMC8210823

[39] Tajima F. Statistical method for testing the neutral mutation hypothesis by DNA polymorphism. Genetics. 1989; 123: 585–595 2513255 10.1093/genetics/123.3.585 PMC1203831

[40] Fu Y-X. Statistical tests of neutrality of mutations against population growth, hitchhiking and background selection. Genetics. 1997; 147: 915–925. 9335623 10.1093/genetics/147.2.915 PMC1208208

[41] Excoffier L, Lischer HEL. Arlequin suite ver 3.5: a new series of programs to perform population genetics analyses under Linux and Windows. Mol Ecol Resour. 2010; 10 (3): 564–567. doi: 10.1111/j.1755-0998.2010.02847.x 21565059 10.1111/j.1755-0998.2010.02847.x

[42] Drummond AJ, Rambaut A, Shapiro B, Pybus OG. Bayesian Coalescent Inference of Past Population Dynamics from Molecular Sequences. Mol Biol Evol. 2005; 22: 1185–1192. doi: 10.1093/molbev/msi103 15703244 10.1093/molbev/msi103

[43] Platt RN, Amman BR, Keith MS, Thompson CW, Bradley RD. What Is *Peromyscus*? Evidence from nuclear and mitochondrial DNA sequences suggests the need for a new classification. J Mammal. 2015; 96: 708–719. doi: 10.1093/jmammal/gyv067 26937047 10.1093/jmammal/gyv067 PMC4668989

[44] Ronquist F. Dispersal-Vicariance analysis: a new approach to the quantification of historical biogeography. Syst Biol. 1997; 46: 195–203. doi: 10.1093/sysbio/46.1.195

[45] Yu Y, Harris AJ, He. S-DIVA (Statistical Dispersal-Vicariance Analysis): a tool for inferring biogeographic histories. Mol Phylogenet Evol. X 2010; 56: 848–850. doi: 10.1016/j.ympev.2010.04.011 20399277 10.1016/j.ympev.2010.04.011

[46] Yu Y, Harris AJ, Blair C, He XJ. RASP (Reconstruct Ancestral State in Phylogenies): a tool for historical biogeography. Mol Phylogenet Evol. 2015; 87: 46–49. doi: 10.1016/j.ympev.2015.03.008 25819445 10.1016/j.ympev.2015.03.008

[47] Rohlf FJ. The tps series of software. Hystrix. 2015; 26:9–12. doi: 10.4404/hystrix-26.1–11264

[48] Martınez JJ, Di Cola V. Geographic distribution and phenetic skull variation in two close species of *Graomys* (Rodentia, Cricetidae, Sigmodontinae). Zool Anz, 2011; 250: 175–194. doi: 10.1016/j.jcz.2011.03.001

[49] Maestri R, Fornel R, Galiano D, de Freitas TRO. Niche Suitability Affects Development: Skull Asymmetry Increases in Less Suitable Areas. PLoS One. 2015; 10(4): e0122412 doi: 10.1371/journal.pone.0122412 25874364 10.1371/journal.pone.0122412 PMC4398368

[50] Rohlf FJ, Slice D. Extensions of the Procrustes method for the optimal superimposition of landmarks. Syst. Zool. 1990; 39: 40–59.

[51] Bookstein FL. Morphometric tools for landmark data: geometry and biology. Cambridge, Cambridge University Press, UK; 1991.

[52] R Core Team. R: a language and environment for statistical computing R Foundation for Statistical Computing, Vienna, Austria; (2014)

[53] Adams DC, Otárola-Castillo E. geomorph: and R package for the collection and analysis of geometric morphometric shape data.–Methods Ecol. Evol. 2013; 4: 393–399. doi: 10.1111/2041-210X.12035

[54] Schlager S. “Morpho and Rvcg—Shape Analysis in R” In: Zheng G, Li S and Szekely G (editors), Statistical Shape and Deformation Analysis, pp. 217–256. Academic Press; 2017.

[55] Klingenberg CP. MorphoJ: an integrated software package for geometric morphometrics. Mol Ecol Res. 2011; 11: 353–357. doi: 10.1111/j.1755-0998.2010.02924.x 10.1111/j.1755-0998.2010.02924.x 21429143

[56] Phillips SJ, Anderson RP, Schapire RE. Maximum entropy modeling of species geographic distributions. Ecol Model. 2006; 190, 231–259. doi: 10.1016/j.ecolmodel.2005.03.026

[57] Elith J, Graham CH, Anderson RP, Dudík M, Ferrier S, Guisan S. et al Novel methods improve prediction of species’ distributions from occurrence data. Ecography. 2006; 29: 129–151. doi: 10.1111/j.2006.0906–7590.04596.x

[58] Peterson AT, Papes M, Eaton M. Transferibility and model evaluation in ecological niche modeling: a comparison of GARP and Maxent. Ecography. 2007; 30: 550–560. doi: 10.1016/j.ecolmodel.2007.11.008

[59] Peterson AT, Papes M, Soberón J. Rethinking receiver operating characteristic analysis applications in ecological niche modeling. Ecol. Model. 2008; 213: 63–72.

[60] Hernandez PA, Graham CH, Master LL, Albert DL. The effect of sample size and species characteristics on performance of different species distribution modeling methods. Ecography. 2006; 29: 773–785. doi: 10.1111/j.0906-7590.2006.04700.x

[61] Swets JA. Measuring the accuracy of diagnostic systems. Science. 1988; 240: 1285–1293 3287615 10.1126/science.3287615

[62] Gent PR, Danabasoglu G, Donner LJ, Holland MM, Hunke EC, Jayne SR et al The Community Climate System Model version 4. J. Climate, 2011; 24: 4973–4991. doi: 10.1175/2011JCLI4083.1

[63] Montes MA, Oliveira LFB, Bonatto SL, Callegari-Jacques SM, Mattevi MS. DNA sequence analysis and the phylogeographical history of the rodent *Deltamys kempi* (Sigmodontinae, Cricetidae) on the Atlantic Coastal Plain of south of Brazil. J Evol Biol. 2008; 26: 1823–1835. doi: 10.1111/j.1420-9101.2008.01586.x 10.1111/j.1420-9101.2008.01586.x 18681917

[64] Quintela FM, Bertuol F, González EM, Cordeiro-Estrela P, Freitas TRO, Gonçalves GL. A new species of *Deltamys* Thomas, 1917 (Rodentia: Cricetidae:) endemic to the southern Brazilian Araucaria Forest and notes on the expanded phylogeographic scenario of *D*. *kempi*. Zootaxa. 2017; 4294: 071–092. doi: 10.11646/zootaxa.4294.1.3

[65] Quintela FM, Gonçalves GL, Bertuol F, González EM, Freitas TRO. Genetic diversity of the swamp rat in South America: Population expansion after transgressive-regressive marine events in the Late Quaternary. Mamm Biol. 2015; 80: 510–517. doi: 10.1016/j.mambio.2015.08.003

[66] Quintela FM, Fornel R, Freitas TRO. Geographic variation in skull shape of the water rat *Scapteromys tumidus* (Cricetidae, Sigmodontinae): isolation-by-distance plus environmental and geographic barrier effects?. An. Acad. Bras. Ciênc. 2016; 88: 451–466. doi: 10.1590/0001-3765201620140631 27142549 10.1590/0001-3765201620140631

[67] Reig OA. Distribuição geográfica e história evolutiva dos roedores muroideos sulamericanos (Cricetidae: Sigmodontinae). *Rev Bras Genet*. 1984; 7: 333–365

[68] Geise L, Smith MF, Patton JL. Diversification in the genus *Akodon* (Rodentia: Sigmodontinae) in southeastern South America: mitochondrial DNA sequence analysis. J Mamm. 2001; 82: 92–101.

[69] Gonçalves PR, Myers P, Vilela JF, Oliveira JA. Systematics of species of the genus *Akodon* (Rodentia: Sigmodontinae) in southeastern Brazil and implications for the biogeography of the Campos de Altitude. Misc. publ.—Mus. Zool., Univ. Mich. 2007; 197: 1–24.

[70] Gonçalves PR, Oliveira JA. An integrative appraisal of the diversification in the Atlantic forest genus *Delomys* (Rodentia: Cricetidae: Sigmodontinae) with the description of a new species. Zootaxa. 2014; 3760:1–38. doi: 10.11646/zootaxa.3760.1.1 24870069 10.11646/zootaxa.3760.1.1

[71] Quintela FM, Gonçalves GL, Althoff SL, Sbalqueiro IJ, Oliveira LFB, Freitas TRO. A new species of swamp rat of the genus *Scapteromys* Waterhouse, 1837 (Rodentia: Sigmodontinae) endemic to *Araucaria angustifolia* Forest in Southern Brazil. Zootaxa. 2014; 3811:207–225. doi: 10.11646/ zootaxa.3811.2.3 10.11646/zootaxa.3811.2.3 24943159

[72] Rull V. Microrefugia. J Biogeogr. 2009; 36: 481–484. doi: 10.1111/j.1365-2699.2008.02023.x

[73] D'Elía G, Pardiñas UFJ. Systematics of Argentinean, Paraguayan, and Uruguayan swamp rats of the genus *Scapteromys* (Rodentia, Cricetidae, Sigmodontinae). J Mammal. 2004; 85: 897–910. doi: 10.1644/BRB-201

[74] D’Elía G, Hanson JD, Mauldin MR, Teta P, Pardiñas UFJ. Molecular systematics of South American marsh rats of the genus *Holochilus* (Muroidea, Cricetidae, Sigmodontinae). J Mammal. 2015; 96: 1081–1094. doi: 10.1093/jmammal/gyv115

[75] Avise JC. Phylogeography: retrospect and prospect. J Biogeog. 2009; 36: 3–15. doi: 10.1111/j.1365-2699.2008.02032.x

[76] Toews DPL, Brelsford A. The biogeography of mitochondrial and nuclear discordance in animals. Mol Ecol. 2012; 21:3907–3930. doi: 10.1111/j.1365-294X.2012.05664.x 22738314 10.1111/j.1365-294X.2012.05664.x

[77] Tomazelli LJ, Villwock JA. Mapeamento geológico de planícies costeiras: o exemplo da costa do Rio Grande do Sul. Gravel. 2005; 3:109–115.

[78] Weschenfelder J, Correa ICS, Aliotta S, Baitelli R. Paleochannels related to late Quaternary sea-level changes in Southern Brazil. Braz J Oceanogr. 2010; 58:35–44. doi: 10.1590/S1679-87592010000600005

[79] Rabassa J, Coronato A, Salemme M. Chronology of the Late Cenozoic Patagonian glaciations and their correlation with biostratigraphic units of the Pampean region (Argentina). J S Am Earth Sci. 2005; 20: 81–103. doi: 10.1016/j.jsames.2005.07.004

[80] Rabassa J, Clapperton C (1990) Quaternary glaciations of the southern Andes. Quaternary Sci Rev. 1990; 9:153–174. doi: 10.1016/0277-3791(90)90016-4

[81] Behling H, Jeske-Pieruschka V, Schüler L, Pillar VP. Campos Sulinos, conservação e uso sustentável da biodiversidade In: Pillar VP, Müller SC, Castilhos ZMS, Jacques AVA, editors. Ministério do Meio Ambiente, Brasília, Brazil, 2009.

[82] Barlow JC. Observations on the biology of rodents in Uruguay. Life sci. contrib., R. Ont. Mus. 1969; 75:1–59.

[83] Quintela FM, Santos MB, Christoff AU, Gava A. Pequenos mamíferos não-voadores (Didelphimorphia, Rodentia) em dois fragmentos de mata de restinga de Rio Grande, Planície Costeira do Rio Grande do Sul. Biota Neotrop. 2012; 12:261–266.

