# Supplementary figures and images for: Pleistocene climatic oscillations in Neotropical open areas: Refuge isolation in the rodent Oxymycterus nasutus endemic to grasslands
Source: PLoS One. 2017 Nov 27;12(11):e0187329. doi: 10.1371/journal.pone.0187329 (PMC5703582; doi:10.1371/journal.pone.0187329)

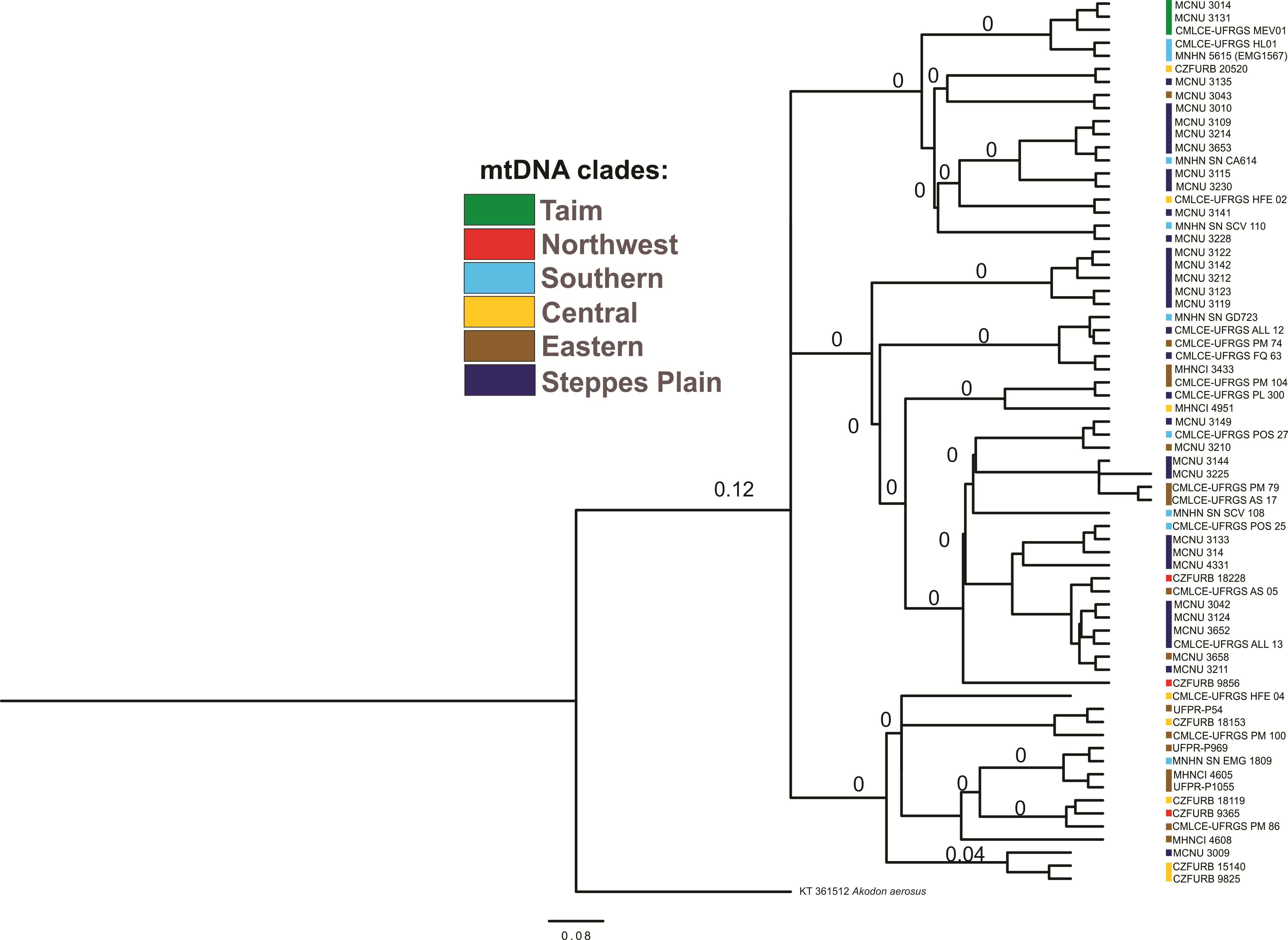

Supplement: S1 Fig — Values above nodes correspond to posterior probabilities > 0.90. (TIF) [file pone.0187329.s001.tif]
