# Supplementary material for: Pleistocene climatic oscillations in Neotropical open areas: Refuge isolation in the rodent Oxymycterus nasutus endemic to grasslands
Source: PLoS One. 2017 Nov 27;12(11):e0187329. doi: 10.1371/journal.pone.0187329 (PMC5703582; doi:10.1371/journal.pone.0187329)
Supplement: S1 Appendix — (DOCX) [file pone.0187329.s004.docx]

**S1Appendix.** Definition of landmarks placed in the three skull views of all *Oxymycterus nasutus* specimens analysed.

Description of landmarks of skull-ventral view:

**1-2:** Longest point parallel between nasal; **3:** Midpoint of upper incisors**; 4-5:** lateralmost point of the alveolus of the incisor; **6-7:** anteriormost margin of incisive foramen; **8-11:** anteriormost margin of zygomaticplate; **9-10:** posteriormost point of suture between premaxilla and maxilla; **12-17:** anteriormost margin of the maximum zygomatic plate posterior constriction; **14-15:** posteriormost margin of incisive foramen; **13-16:** anteriormost margin of first molar alveolus; **18-20:** posteriormost margin of third molar; **19:** posteriormost point of suture between palatines; **21-22:** posteriormost margin of the maximum anterior constriction of squamosal root of zygomatic;  **23-24:** posterior end of squamosal root of zygomatic bar; **25-26:** basis length of suture between basisphenoid and basiocciptal; **27-28:** superiormost margin of tympanic bulla (ectotympanic);  **29-31:** midpoint margin of occipital condyle; **30:** posteriormost point of superior margin of foramen magnum; **32:** anteriormost point of inferior margin of foramen Magnum.

Description of landmarks of skull dorsal view:

**1:** anteriormost point of suture between nasals; **2-3:** anteriormost point of suture between nasal and premaxilla; **4-5:** anteriormost point of zygomatic plate; **6-7:** anteriormost margin of the maximum constriction of squamosal root of zygomatic arch; **8-9:** superiormost point of suture between parietal and occipital;  **10:** posteriormost point of occipital margin; **11:** suture between parietals and interparietal; **12:**  suture between frontals and parietals; **13:** suture between nasals and frontals;  **14-15:** length margim of maximum constriction of interorbital region (frontal); **16-17:** posteriormost margin of maximum constriction of antorbital bridge.

Description of landmarks of skull lateral view:

**1:** anteriormost point of nasal; **2:** anteriormost point of the suture between the nasal and the premaxilla; **3:** posteriormost point of incisor alveolus; **4:** inferiormost point of incisor alveolus; **5:**  ventral extent of infraorbital foramen; **6:** point of maximum posterior constriction of antorbital bridge; **7:** anteriormost point of the molar row; **8:** posteriormost point of the molar row; **9**: point of maximum anterior constriction of squamosal root of zygomatic arch; **10:** anteroventral limit of the tympanic bulla;  **11:** ventralmost point at the middle of the tympanic bulla;  **12:** inferior extremity on the boundary between the occipital condyle and the tympanic bulla; **13:** curvature at the limit between the occipital condyle and the occipital bone; **14:** suture between parietal, squamosal and occipital; **15:** superiormost point of suture between pariental and interparietal; **16:** superiormost point of suture between frontal and parietal.
