# Supplementary material for: Pleistocene climatic oscillations in Neotropical open areas: Refuge isolation in the rodent Oxymycterus nasutus endemic to grasslands
Source: PLoS One. 2017 Nov 27;12(11):e0187329. doi: 10.1371/journal.pone.0187329 (PMC5703582; doi:10.1371/journal.pone.0187329)
Supplement: S2 Appendix — (DOCX) [file pone.0187329.s005.docx]

**S2 Appendix.** Table Additional localities of *Oxymycterus nasutus* recover by Literature for Spatial Distribuition Modelling.

*****Geographical coordinates were estimated based on the municipality and/or location available.

| **Reference** | **Country** | **State/Dept** | **Municipality/Locality** | **Lat. (S), Long (W).** |
| --- | --- | --- | --- | --- |
| Grazzini et al. 2015 | BR | PR | Piraí do Sul, Floresta Nacional de Piraí do Sul | -24.577163, -49.918777 |
| Oliveira and Gonçalves 2015 | UR | Canelones | *Salinas, Arroyo Tropa Vieja | -34.775663, -55.875237 |
| Oliveira and Gonçalves 2015 | UR | Cerro Largo | *Melo, 6km ao SE | -32.414957, -54.133716 |
| Oliveira and Gonçalves 2015 | UR | Rocha | *Lascano, 22km ao SE | -33.790417, -54.068686 |
| Oliveira and Gonçalves 2015 | UR | Maldonado | *San Carlos, 15km ao Norte | -34.751549, -54.924380 |
| Hoffmann et al. 2002 | UR | Cerro Largo | *Rio Tacuari, 20 km SE Melo | -32.519200, -54.028395 |

**References**

Grazzini G, Mochi-Junior CM, Oliveira H, Pontes JS, Gatto-Almeida F, Tiepolo LM. Identidade, riqueza e abundãncia de pequenos mamíferos (Rodentia e Didelphimorphia) de área de Floresta com Araucária no estado do Paraná, Brasil. Papéis Avulsos de de Zoologia (MZUSP). Volume 55(15):217‑230, 2015. <http://dx.doi.org/10.1590/00311049.2015.55.15>.

Oliveira JA, Gonçalves PR (2015) Suborder Myomorpha: Family Cricetidae: Subfamily Sigmodontinae. Genus *Oxymycterus*; pp. 247–268 in: J.L. Patton, U.F.J. Pardiñas and G. D’Elía (eds.). Mammals of South America 2: Rodents. Chicago: University of Chicago Press. doi: 10.7208/chicago/9780226169606.001.0001

Hoffmann, F.G., E.P. Lessa and M.F. Smith. 2002. Systematics of *Oxymycterus* with description of a new species from Uruguay. Journal of Mammalogy 83(2): 408–420. doi: 10.1644/1545-1542
