# Supplementary material for: Pleistocene climatic oscillations in Neotropical open areas: Refuge isolation in the rodent Oxymycterus nasutus endemic to grasslands
Source: PLoS One. 2017 Nov 27;12(11):e0187329. doi: 10.1371/journal.pone.0187329 (PMC5703582; doi:10.1371/journal.pone.0187329)
Supplement: S1 Table — (DOCX) [file pone.0187329.s006.docx]

**Table S1.** Genetic divergence (using *p-*distance) between pairs of *Cytb* haplotypes recovered from different clades of *O. nasutus* determined by the Bayesian phylogeny.

| **Clades** | **Northwest** | **Central** | **Eastern** | **Coastal Plain** | **Southern** | **Within mean group distance** |
| --- | --- | --- | --- | --- | --- | --- |
| Northwest | - |  |  |  |  | 0.009 |
| Central | 0.016 | - |  |  |  | 0.002 |
| Eastern | 0.013 | 0.010 | - |  |  | 0.003 |
| Steppes Plain | 0.016 | 0.012 | 0.012 | - |  | 0.004 |
| Southern | 0.019 | 0.014 | 0.016 | 0.015 | - | 0.004 |
| Taim | 0.023 | 0.020 | 0.020 | 0.021 | 0.025 | 0.004 |
