# Supplementary material for: Pleistocene climatic oscillations in Neotropical open areas: Refuge isolation in the rodent Oxymycterus nasutus endemic to grasslands
Source: PLoS One. 2017 Nov 27;12(11):e0187329. doi: 10.1371/journal.pone.0187329 (PMC5703582; doi:10.1371/journal.pone.0187329)
Supplement: S2 Table — (DOCX) [file pone.0187329.s007.docx]

Table S2. Percentage of correct classification from discriminant analysis, using Jackknife Cross-validation, for form in dorsal, ventral, and lateral view of the skull for two Ecorregions where is inserts the *Oxymycterus nasutus.*

| Groups | Predicted group membership | | |
| --- | --- | --- | --- |
|  | Pampas | Atlantic Forest | Overall % |
| Dorsal view of skull |  |  |  |
| Pampas (N=51) | 94.1176 | 5.8824 | 94.11 |
| Atlantic Forest (N=38) | 7.8947 | 92.1053 | 92.10 |
|  |  |  |  |
| Ventral view of skull |  |  |  |
| Pampas (N=50) | 82 | 18 | 82 |
| Atlantic Forest (N=38) | 17.949 | 82.051 | 82.05 |
|  |  |  |  |
| Lateral view of skull |  |  |  |
| Pampas (N=50) | 86 | 14 | 86 |
| Atlantic Forest (N=38) | 25.641 | 74.359 | 82.05 |
