# Supplementary material for: Pleistocene climatic oscillations in Neotropical open areas: Refuge isolation in the rodent Oxymycterus nasutus endemic to grasslands
Source: PLoS One. 2017 Nov 27;12(11):e0187329. doi: 10.1371/journal.pone.0187329 (PMC5703582; doi:10.1371/journal.pone.0187329)
Supplement: S3 Table — (DOCX) [file pone.0187329.s008.docx]

**S4 Table.** Percentage of correct classification from discriminant analysis, using Jackknife Cross-validation, for form in dorsal, ventral, and lateral view of the skull for phylogenetic clades of *Oxymycterus nasutus*.

| **Dorsal View** | Southern | Central | Steppes Plain | Taim | Eastern | Northwest | Overall |
| --- | --- | --- | --- | --- | --- | --- | --- |
| Southern | 50 | 0 | 50 | 0 | 0 | 0 | 50 |
| Central | 0 | 47.3684 | 15.7895 | 0 | 31.5789 | 5.2632 | 47.36 |
| Steppes Plain | 2.3256 | 4.6512 | 86.0465 | 4.6512 | 2.3256 | 0 | 86.04 |
| Taim | 50 | 0 | 50 | 0 | 0 | 0 | 0 |
| Eastern | 0 | 43.75 | 0 | 0 | 50 | 6.25 | 50 |
| Northwest | 0 | 66.6667 | 0 | 0 | 33.3333 | 0 | 0 |
|  |  |  |  |  |  |  |  |
| **Ventral View** | Southern | Central | Steppes Plain | Taim | Eastern | Northwest | Overall |
| Southern | 25 | 0 | 75 | 0 | 0 | 0 | 25 |
| Central | 0 | 61.9048 | 14.2857 | 9.5238 | 4.7619 | 9.5238 | 61.90 |
| Steppes Plain | 0 | 16.6667 | 78.5714 | 2.38 | 0 | 2.3810 | 78.57 |
| Taim | 25 | 25 | 50 | 0 | 0 | 0 | 0 |
| Eastern | 0 | 13.3333 | 0 | 0 | 73.3333 | 13.3333 | 73.33 |
| Northwest | 0 | 66.6667 | 0 | 0 | 33.3333 | 0 | 0 |
|  |  |  |  |  |  |  |  |
| **Lateral View** | Southern | Central | Steppes Plain | Taim | Eastern | Northwest | Overall |
| Southern | 25 | 0 | 25 | 25 | 0 | 25 | 25 |
| Central | 4.7619 | 47.6190 | 14.2857 | 0 | 28.5714 | 4.7619 | 47.61 |
| Steppes Plain | 0 | 2.3810 | 76.1905 | 7.1429 | 14.2857 | 0 | 76.19 |
| Taim | 0 | 0 | 75 | 25 | 0 | 0 | 25 |
| Eastern | 0 | 40 | 26.6667 | 0 | 33.3333 | 0 | 33.33 |
| Northwest | 0 | 100 | 0 | 0 | 0 | 0 | 0 |
